# Reproductive immunology in viviparous mammals: evolutionary paradox of interactions among immune mechanisms and autologous or allogeneic gametes and semiallogeneic foetuses

**DOI:** 10.1080/01652176.2020.1852336

**Published:** 2020-12-07

**Authors:** M. Samardžija, M. Lojkić, N. Maćešić, H. Valpotić, I. Butković, J. Šavorić, I. Žura Žaja, D. Leiner, D. Đuričić, F. Marković, P. Kočila, Z. Vidas, M. Gerenčer, A. Kaštelan, A. Milovanović, M. Lazarević, D. Rukavina, I. Valpotić

**Affiliations:** a Clinic for Obstetrics and Reproduction of Animals, Veterinary Faculty University of Zagreb, Zagreb, Croatia; b Department for Animal Nutrition and Dietetics, Veterinary Faculty University of Zagreb, Zagreb, Croatia; c Department for Physiology and Radiobiology, Veterinary Faculty University of Zagreb, Zagreb, Croatia; d Department of Anatomy, Histology and Embriology, Veterinary Faculty University of Zagreb, Zagreb, Croatia; e Veterinary Practice, Đurđevac, Croatia; f Belupo d.d. Danica, Koprivnica, Croatia; g Animal Feed Factory, Čakovec, Croatia; h Faculty of Medicine, Department of Urology, Josip Juraj Strossmayer University of Osijek, Osijek, Croatia; i Croatian Academy of Sciences and Arts, Croatian Academy of Sciences and Arts, Zagreb, Croatia; j Department of Reproduction, Veterinary Scientific Institute, Novi Sad, Serbia; k Department for Physiology and Biochemistry, Faculty of Veterinary Medicine, University of Belgrade, Belgrade, Serbia; l Department of Cellular Immunology, Baxter Hyland Immuno, Vienna, Austria; m Department of Biology, Veterinary Faculty University of Zagreb, Zagreb, Croatia

**Keywords:** Reproductive immunology, transplantation immunology, viviparous mammals, semiallogeneic foetus, allogeneic gamete, immunotrophism, CD45

## Abstract

Literally, reproductive immunology was born in bovine on-farm reproduction where seminal experiments intended for developing methods for embryo transfer in cattle were performed. Actually, these experiments led to two of major concepts and fundamental principles of reproductive immunology using the bovine species as a model for biomedical research, namely the concept of acquired immunological tolerance and the paradox of the semiallogeneic bovine foetus whereby such organism can develop within an immunologically competent host. Peter Medawar, a scientist who together with Frank Macfarlande Burnet shared the 1960 Nobel Prize in physiology or medicine for discovery of acquired immunological tolerance, while studying dizygotic cattle twins, thereby giving birth to reproductive immunology. Also, these findings significantly influenced development of organ transplants and showed that using farm animals as models for studying transplantation immunology had general relevance for mammalian biology and health including those of humans. However, the interest for further research of the fascinating maternal immune influences on pregnancy and perinatal outcomes and of the prevention and treatment of immunologically mediated reproductive disorders in viviparous mammals of veterinary relevance by veterinary immunologists and reproductive clinicians have been very scarce regarding the application of nonspecific immunomodulatory agents for prevention and treatment of subfertility and infertility in pigs and cattle, but still broadening knowledge in this area and hold great potential for improving such therapy in the future. The aim of the current overview is to provide up-to-date information and explaining/translating relevant immunology phenomena into veterinary practice for specialists and scientists/clinicians in reproduction of animals.

## 1. Introduction

The purpose of this overview is to present the origins, scope, and some of the achievements of research in reproductive immunology, but also to indicate the significant impact of its authentic discoveries that had on the development of human and veterinary immunology in general. Namely, immunological aspects of animal reproduction, particularly its efficiency in farm viviparous mammals have been designated as controversial in regard that a female tolerated the intrusion of male spermatozoa, *i.e.,* allogeneic cells and persisting presence of semi allogeneic foetus, while reserving the prerogative of rejecting allogeneic grafts of tissue. Several theories were proposed to explain this paradox, each of which failed to withstand careful examination considering the fundamental aspects of reproductive immunology and its clinical application as follows: (1) astonishingly complex mechanisms of immune system function, (2) delicate adaptations to placentation, (3) unique compartmentalization of the reproductive tract from systemic immunity, (4) impact of endocrine influences, (5) proper selection of relevant experimental animal models and (6) poorly designed and/or validated observational trials.

This overview brings the current state of research in reproductive immunology in humans and other mammals, with particular emphasis of its emerging importance relevant to veterinary science. The aim of this review was to provide readers with understandable main research activities and evidence-based clinical practice potentials in the field of veterinary reproductive immunology. Namely, it is certain that this field will clearly be a future source of data for significant improvement in the prevention and treatment of a variety of reproductive disorders of domestic mammals by explaining and translating the critical roles that immune factors play in reproduction to the reproductive specialists. Such approach and the significant historical contributions to the field provide the introductory resource for both veterinary clinicians and scientists.

## 2. Reproductive immunology in viviparous mammals: past, present and future

Reproductive immunology became a research focus in biomedical sciences about 70** **years ago as concepts regarding transplantation tissue antigens and tissue transplant rejection have been discovered. A pioneering 1953 paper that fundamentally influenced development of this subspecialty of immunology was „Some immunological and endocrinological problems raised by the evolution of viviparity in vertebrates “(Medawar 1953). His concepts of foetal allograft and results of studying dizygotic bovine twins (Anderson et al. 1951) followed by transplantation of tissue between foetal mice without rejection (Billingham et al. 1953) had significant impact on development of organ transplants. Besides, his ideas and success stimulated further intensifying of basic research using mammalian species of domestic farm animals not only regarding their health and productivity but also to improve understanding of mammalian biology (Billingham and Beer 1984). A key observation of Medawar that immunologic tolerance could be induced by histocompatibility antigens exposure during foetal life so that adults tolerate expression of these antigens that they were exposed to while foetuses, profoundly influenced the areas of reproductive immunology and immunogenetics relevant to transplantation immunology (Medawar 1961) with a final aim to enhance human health (Hansen 2010).

The triad of individuals involved in viviparous mammalian reproductive processes (mother/female, father/male, descendants/offspring), the dynamic and rapidly changing aspects of the maternofoetal (M-F) interface during development, the unique properties of reproductive tract immune cells and the lack of specimen availability during mid to late pregnancy in humans and wildlife, due to the desire of healthy offspring make reproductive immunology a challenging subject.

However, its importance to every current, past and future mammal, cannot be overstated. Rather than addressing the mammalian conceptus as an allograft, parasite or transplant to be tolerated, appreciation of the unique immunological features of mammalian reproduction will be the approach most likely to advance translation of research in this field (Croy 2014a). Thus, special research topics on reproductive immunology are more clinically oriented and address current understanding of the immunological aspects of common pathologies that complicate human and livestock reproduction. In the contemporary framework, immune system promotion of reproductive success (immunotrophism) is strongly supported, whereas immune system-induced pathologies and failures of mammalian reproduction provide key opportunities to understand the disruption of homeostasis (Croy 2014b). Today it is well known that the immune system plays a critical role in pregnancy establishment and maintenance, but application of immunological principles to the treatment of infertility and pregnancy loss has had inconsistent outcomes leading to scepticism and misinterpretation of data (Young 2016).

Future improvement of knowledge in the area of reproductive immunology will clearly be a promising basis for meaningful and better prevention and treatment of many reproductive disorders and subfertility in animal and human patients. Moreover, the major histocompatibility system (MHS) or complex (MHC) of polymorphic and multiallelic gene clusters controlling the expression and reactivity of both tissue and leukocyte antigens in animals and humans is not considered to be only of importance in transplantation immunology, due to the fact that it might also control specific immune responsiveness in vertebrates. In veterinary relevant species the association between transplantation -, tissue - histocompatibility - or leukocyte/lymphocyte antigens, and their homology (Medawar 1946) may offer valuable practical approaches to improve animal breeding and health programs, particularly in livestock production.

Instead of any conclusions, just a reminding note that reproductive immunology is a complex field of general immunology, of priority importance for species survival and in numerous aspects also in human and veterinary medicine, with many connections to the other areas of life sciences and great potential for immunotherapy.

### 2.1. A little of evolutionary biology and history

It is assumed that evolution of the immune system began more than 600 million years ago (mya) when appeared ancestral forms of: (1) the MHC genes and their products (1.1.) the transmembrane tissue-antigens displayed on cell membranes of all self-cells, (1.2.) the intracellular peptides allowing T lymphocytes to bind to, either recognize, and tolerate self (auto recognition) or unrecognised, and intolerate non self (allorecognition) following presentation as potential foreign antigens to their T cell receptor (TCR) and (2) the gene encoding an enzyme, protein tyrosine phosphatase, receptor type C (PTPRC), also known as the cluster of differentiation (CD45) antigen, displayed exclusively on membrane of leukocytes establishing innate immunity.

By 500 mya, the evolution of recombination-activating genes (*Rag*) had occurred in fish, founding adaptive immunity. The *Rag* genes encode parts of a protein complex that plays important roles in the recombination of the genes encoding immunoglobulin (Ig) and TCR molecules. There are *Rag* 1 and *Rag* 2 genes, whose cellular expression is restricted to lymphocytes during their developmental stages. The enzymes encoded by these genes are essential to the generation of mature B and T cells that are crucial components of the adaptive immune system. Unique characteristic of this system shared by all vertebrates (except agnathans) is a highly diversified repertoire of antigen receptors encoded by the Ig and TCR gene loci. In order to encode functional protein receptors the genes within these loci evolve by their assembly from an array of individual *V, D* and *J* gene segments into functional antigen receptor genes in a strictly controlled, site-specific process termed *V (D) J* recombination of conserved DNA sequence (Fugmann 2010). However, antigens are not the only key regulators of immune cell biology in the reproductive tract due to the fact that endocrine influences are critical. Further, reproductive immunology addresses not only pregnancy, but the reproductive tracts of males and non-gravid females. The many organs that comprise each of the male and female reproductive systems are components of the mucosal immune system. Each is a specialized environment that makes unique contributions to the success of reproduction and can experience immunologically mediated inflammatory diseases. Such diseases, including autoimmune disorders are predisposed genetically and/or environmentally and may be triggered by dysregulation of the normal immune response, and thus have clinical importance not only for humans (Kutteh et al. 2019), but also for food mammalian species in livestock production (Croy 2014a).

The first mammals appeared much later (220 mya) and reproduced by laying eggs. Placental mammals with internal gestations and live born offspring have existed for only 100 mya, with mice and humans being relatively recent mammalian evolutions. This historical record indicates that the evolution of viviparity must have involved processes that were immunologically unrecognized, immunologically accepted or occurred at accelerated rates that could not be immunologically regulated. Placental evolution occurred repeatedly and is not regarded as increasing genetic changes but as revolutionary sudden changes. Current research suggests acquisition of ancient endogenous retroviruses that drove cell fusion was critical for evolution of placental mammals. Consequently, placenta has the most diverse histological structure among species of any tissue. It is not yet known in detail between species (Croy 2014a). However, it is quite clear that immune cell populations of innate immunity have been specially adapted to this location in all species studied to date. Whether or not immunological specializations at the M-F interface also vary widely is not fully elucidated yet. Moreover, the evolution of placental function has been accompanied by formation of new genes during recent evolution so that orthologues actually do not exist in any but closely related species.

Historically, farm animal species utilization as models for biomedical research were shown to be important for the development of the field of reproductive immunology (Hansen 2010). It is a logical approach that a diverse array of genotypes are used to study immunological principles relevant to mammalian biology and human health. Due to the fact that the nature of mammalian evolution has resulted in a situation where the genomes of the most commonly used animal models, the laboratory rodents, are less related to the human genome than those of domestic farm ungulates, such as swines, cattle, sheep, and horses, (Hansen 2011). Although, the common ancestor of these animals diverged from humans before the common ancestor of rodents and humans, physiologically and immunologically characteristics of their genomes are more similar to the human genome than are the genomes of laboratory rodents, such as: mice, rats, guinea pigs, hamsters (from order: *Rodentia*) and rabbits (from order: *Lagomorpha*). Domestic farm animals and horses are not the only mammalian species that can be useful models in biomedical research, but they offer advantages: of availability, ease of handling, low cost partly due to utilization as food animals, and of traditionally well-known biology and a well-described husbandry, as well as of the acceptance as a models by the scientific community.

### 2.2. Key discoveries and current knowledge

Directly or indirectly, immunology has now intruded into, or been shown to underlie, nearly every aspect of mammalian reproduction. It may be the basis of a natural defence mechanisms or of a disease processes, it may afford an approach to the artificial control of certain reproductive failures, it may provide a novel approach to an investigation, and it may be the basis of a clinical assay procedure. Thus, this part of the overview is designed to provide a comprehensive summary of the key achievements and of what is of essence known about the reproductive immunology in humans and animals of veterinary relevance. We shall also indicate the significant impact of certain discoveries, particularly the role of the MHC in reproductive and transplantation immunology on the development of immunology in general.

It is noteworthy to say, that in the period from 1960 and 1996 four Nobel Prizes were shared in Physiology or Medicine for the discoveries within the area of reproductive immunology, and it’s biologically closely related, but more clinically oriented fields of transplantation immunology and immunogenetics. In the 1960, Peter B. Medawar was awarded his Nobel Prize with Frank M. Burnet for their work in tissue grafting which is the basis of organ transplants, and their discovery of „acquired immunological tolerance“. Furthermore, George D. Snell shared the 1980 Nobel Prize in Physiology or Medicine with Baruj Benacerraf and Jean Dausset for their discoveries concerning "genetically determined structures on the cell surface that regulate immunological reactions". They specifically "discovered the genetic factors that determine the possibilities of transplanting tissue from one individual to another,” and introduced the concept of H antigens “by discovery of the human leukocyte antigens (HLA), the MHC, in humans and all vertebrates. Medawar's discovery resulted in a shift of emphasis in the science of immunology from one that attempts to deal with the fully developed immunity to one that attempts to suppress the body's rejection of organ transplants. It directly laid the foundation for the first successful organ transplantation in humans, in 1954 by Joseph E. Murray, who jointly received the 1990 Nobel Prize in Physiology or Medicine with Donnall E. Thomas. Tthe Nobel Prize in Physiology or Medicine for 1996 was awarded jointly to Peter C. Doherty and Rolf M. Zinkernagel "for their discoveries concerning the specificity of the cell mediated immune defence." They demonstrated in 1974, that the MHC is also involved in the regulation of the cellular cytolytic reaction to virus-infected isogeneic cells or tissue. Therefore, they postulated that the principle of simultaneous „dual recognition “is essential for the ability of the immune system to distinguish between ‘self’ and ‘non-self’ (Götze 2012).

However, it will be necessary to find more details on some of these achievements and potentialities of reproductive immunology, which could not be presented herein due to the fact that the issues addressed in this overview are broad, and the text of this journal is limited. Thus, readers are advised to refer to the related high quality original articles, reviews and book chapters cited below (Gerenčer et al. 1979; Van Dam 1981; Gerenčer and Kaštelan 1983; Billingham and Beer 1984; Gerenčer et al. 1988; Lazarević et al. 1995; Ober and Van Der Ven 1997; Rukavina et al. 1998; Lazarević et al. 2003; Blois et al. 2007; Laškarin et al. 2007; Hansen 2010, 2011; Dominović and Rukavina 2011; Makrigiannakis et al. 2011; Götze 2012; Veljković-Vujaklija et al. 2012; Christiansen 2013; Redžović et al. 2013; Yitbarek and Regasa 2014; Young 2016; Kutteh et al. 2019; Osborne et al. 2019) regarding key discoveries in this highly complex immunological paradox such as mammalian pregnancy, in which foetal semiallograft manage to survive in a potentially hostile environment, genetically different in a part, but fully immunocompetent.

## 3. Immunological phenomena inducing clinical infertility and conceptus failure in mammals

Biomedical animal research is almost totally murine oriented, using the other rodent models to a lesser extent (Hansen 2010). Undoubtedly, the laboratory mouse has proven to be an invaluable model for biological/immunological research, and the most of what we know today about mammalian biology is derived from research performed with *Mus musculus*. However, to reject the other animal models would be to ignore the essential need to address evolutionary divergence among mammals by studying, in particular reproductive immunology/immunogenetics using an array of species/genotypes. For example, the intriguing insights in the nature and development of acquired immunological tolerance have been postulated because Medawar recognized the unique properties of the placenta vasculature of dizygotic twin calves (Anderson et al. 1951). Historically, farm animal models were important for the development of the field of pregnancy immunology (Hansen 2010, 2011). In particular, in domestic ungulates, the importance of interactions between the cells and molecules of the immune system with the conceptus during early pregnancy, *i.e.,* from the period of semen deposition through initial apposition and interdigitating of the trophoblast with the endometrial epithelium. During this period, the immunological situation changes from the cleavage stages of development, when the conceptus apparently evades detection by the maternal immune system, to a time coincident with trophoblast elongation and initial placentation, when large‐scale changes in endometrial function are brought about by conceptus cytokine secretion or alloantigen expression. Interactions between components of the maternal immune system and the conceptus can be either beneficial or harmful. In particular, development and differentiation can be promoted by specific cytokines, but the activation of cell‐mediated immunity against the conceptus or inflammatory and immunological events in response to infectious disease can lead to the demise of the conceptus. Moreover, immune responses generated outside the reproductive tract can also compromise fertility.

The best studied example is mastitis in dairy cattle. Several studies in cows which have been classified based on the presence of clinical or subclinical mastitis indicate that occurrence of mastitis is associated with reduced establishment of pregnancy and increased pregnancy loss (Hansen et al. 2004). The mechanism by which an immune response in one region of the body, such as mammary gland causes changes in the reproductive tract is likely to involve release of bacterial products, cytokines and chemokines from the site of infection/inflammation that affect (i) the hypothalamic - pituitary axis to reduce gonadotropin releasing hormone (GnRH) and luteinizing hormone (LH) release, (ii) at the ovary to reduce progesterone (P4) secretion, and (iii) at the reproductive tract to block embryonic development (Hansen 2011). The later review article of this author highlights additional examples whereby domestic farm animals are being used to develop concepts pertinent to a wide range of mammalian species and humans. As shown through more recently published reviews/book chapters (Yitbarek and Regasa 2014; Kutteh et al. 2019; Osborne et al. 2019), domestic companion and farm animals are providing important insights into the nature of the conceptus-maternal immunological relationship, hormonal regulation of uterine function, host defence mechanisms in the reproductive tract, role of endogenous retroviruses in placentation and involvement of the immune system in function of the *corpus luteum.*

However, to know and understand more about the reproductive immunology of animals of veterinary relevance as well of humans including their clinical applications and impacts, the current overview is made from different sources which can be consulted by referring to the abovementioned references (and their literature) as well as to all other references cited herein.

The term reproductive immunology covers the area of immunology relating to all stages of the process of reproduction: (i) gametogenesis, (ii) fertilization of gametes, (iii) implantation of embryos in the uterus, (iv) invasion and development of the placenta in early and late pregnancy and (v) parturition. All these stages of mammalian reproduction are associated with autoimmune factors frequently inducing the immune-mediated endocrinopathies or autoimmune diseases/syndromes. Several autoimmune factors have been investigated as potential influences on reproductive success and failure. Despite decades of intensive research, little is known about the distinct impact of these factors on pregnancy outcome in viviparous mammals. Controversy still exists concerning „history of life “that for a pregnancy to succeed, two immunologically and genetically different tissues must coexist for several weeks or months. In fact, we are just beginning to understand the complex interactions between the endocrine and immune systems that support the foetal semi-allograft and, thus, sustain continuation of mammalian species reproduction.

Rather than addressing the mammalian conceptus as an allograft to be tolerated, appreciation of the unique immunological features of mammalian reproduction will be the approach most likely to advance translation of research in this field. Life history theory postulates that, as long as survival is assured, limited resources are available for reproduction, maintenance, and growth/storage. To maximize lifetime reproductive success, resources are subject to trade-offs both within individuals and between current and future risk.

### 3.1. Males produce autoantibodies against autologous sperm cells

Immune responses to self-antigens generally represent a failure of immunological tolerance. Autoimmune responses against autologous sperm cells are believed to occur because certain T-helper cells are induced or critical suppressor cells are inhibited. As a result, the ‘forbidden’ clones of T and B cells bearing receptors for self-antigens emerge and expand, leading to the production of autoantibodies, cytotoxic T lymphocytes and inflammatory T cells directed at self-antigens expressed by spermatozoa. Autoimmunity to sperm cells is manifested in a variety of clinical conditions which all include infertility. These are as follows: autoimmune orchitis that is the consequence of an immune response to testicular antigens; idiopathic infertility in males and females that is frequently associated with autoantibodies formation to autologous sperm cells, and vasectomy that is often followed by autoimmune pathology in the testes (Raghupathy et al. 1990). The most likely explanation is that the sperm antigens appear long after immunocompetence develops, and long after immunological tolerance to other self-antigens is established, and thus, they are recognized as foreign. Furthermore, the testis and residing sperm cells are separated from the immune system by a blood-testis barrier which is normally intact. However, if the testis become accessible to the immune system (like after vasectomy), its antigens would be recognized as non-self, resulting in an autoimmune reaction. Such thesis have been argumented by the observation that some testicular autoantigens (recognizable by antibodies either produced by an active immunization or passively administered) are found outside the blood-testis barrier and are exposed to the immune system It has been demonstrated also that the passive transfer of activated T cells specific for sperm antigens obtained from mice with induced experimental autoimmune orchitis into normal mice resulted in the development of autoimmune disease (Mahi-Brown et al. 1987). These T cells reacted with autoantigens in the *vas deferens*, *rete testis* and the straight tubules connecting the *seminiferous* tubules with the *rete testis*, suggesting that the blood-testis barrier is not totally intact.

### 3.2. Females produce alloantibodies against allogeneic spermatozoa

Porcine and bovine sperm cells are antigenic in females as reported by Vaiman et al. (1978) and Tripathi et al. (1999), respectively. Spermatozoa are antigenic when injected subcutaneously into females, and isoimmunisation with semen and testis can lead to infertility in cattle (Menge 1967). Following mating, spermatozoa from the reproductive tract must be removed without development of humoral or cellular immune responses to sperm cells. Indeed, deposition of sperm into the reproductive tract leads to an inflammatory response characterized by influx of phagocytic cells (neutrophils, macrophages, and dendritic cells) and T lymphocytes as well as increased local production of cytokines such as granulocyte-macrophage colony stimulating factor 2 (CSF2), IL-6 and monocyte chemotactic protein-1 (Schuberth et al. 2008). The most well studied of these cytokines with respect to their pro-developmental actions on the embryo in humans, mice, and pigs is CSF2. Also, it inhibits embryo apoptosis as reported for mice and cattle (Loureiro et al. 2011a, 2011b). It is likely that the inflammatory response to semen contributes to the removal of sperm by the innate immune system, particularly by phagocytosis of sperm cells mainly by neutrophils, and thereby prevents adaptive immune system responses against sperm cells (Robertson et al. 2009; Hansen 2011). Human spermatozoa contain antigens that are foreign to both male and female immune systems. Sperm alloantibodies that are bound to spermatozoa could be found in over half of seminal fluid and/or serum samples tested after vasectomy as during this procedure, the blood-testis barrier is broken and sperm antigens are exposed to the immune cells and molecules. Thus, the sperm cells-bound antisperm antibodies (ASA) can be found to persist after vasectomy, but not in all cases result in failure of fertilization. The aetiology of sperm-directed immunity in females is largely unknown. However, the possible mechanisms include cross-reactivity with microbial antigens, violation and sensitization at intestinal mucosal surfaces, and interferon gamma (IFN γ) – mediated potentiating of the antisperm immune response in females whose male partners have sperm autoantibodies in their semen (Clarke 2009). It has been suggested that the ASA are a cause of unexplained infertility in women. Sperm immobilization, inhibition of cervical mucus penetration, and interference with processes that lead to sperm-oocyte binding are some of the mechanisms by which ASA hamper fertilization. Further, it has been proposed that ASA interfere with normal pregnancy by inhibition of implantation of the early embryo (Marshburn and Kutteh 1994).

However, it is now recognized that not all ASA interfere with fertilization or impair fertilization at the same level. Thus, a more practical approach regarding the prognostic value of the ASA screening for diagnosis of infertility, would be to first identify antibodies that bind to specific antigens that impair such delicate processes as the acrosomal reaction (Kutteh et al. 2019).

### 3.3. Females may develop autoimmunity to their own gonadal material

Substantial evidence indicates that ovarian insufficiency can result from autoimmune processes directed against the steroidogenic cells of the ovarian follicle (Kutteh et al. 2019). Ovarian histopathological studies in steroid cell antibody positive patients with premature ovarian failure consistently show lymphocytic oophoritis with infiltrate predominantly populated by CD4^+^ and CD8^+^ T cells and plasma cells, indicating the presence of one among the other destructive autoimmune endocrinopathies causing infertility (Kutteh 1996). There are several other autoimmune diseases/syndromes associated with pregnancy in humans as referred by Kutteh et al. (2019) which are described in more detail regarding their immune aetiology and histocytopatogenesis. Also, there are numerous studies attempted to identify autoantibodies in females associated with pregnancy loss and infertility. The most commonly studied include antiphospholipid (APL) and antithyroid antibodies (ATA). Antiovarian antibodies have been associated with polyglandular autoimmune syndromes type I and type II. Other autoantibodies of potential interest to reproduction include antiligand (gliadin) antibody (AGA), antinuclear antibodies (ANA), ASA, and endometriosis-associated immune responses (Cervera and Balasch 2010). For more information on this issue refer to Kutteh et al. (2019).

### 3.4. Foetomaternal relationships: immune tolerance or incompatibility and abortion

It is still not elucidated how the foetal-placental unit (often called the semiallogenic foetal graft) in most instances evades rejection by the maternal immune system. However, it has become increasingly clear that the M-F interface is not an immunologically inert area but a series of active immune processes, some of which have specificity for foetal antigens, taking place at the maternal side of the trophoblast during pregnancy (Christiansen 2013). Although somewhat different from original ideas postulated almost 7 decades ago, by Medawar and collaborators (Billingham et al. 1953), the mechanisms of foetomaternal immunotolerance are now thought to involve the spatiotemporally coordinated effects of numerous hormonal and immunogenetical/immunological factors that modify the maternal immune system’s response to the semiallogeneic conceptus (Makrigiannakis et al. 2011). Additionally, the immune, endocrine and neurological interactions between mother and foetus are bidirectional and even more complex than can be compared with a tissue allograft. Namely, despite the well-known fact that the foetus harbours paternal and maternal alloantigen’s, and thus is more precisely called semi-allograft still must avoid potentially hostile alloimmunity. The mechanisms that could account for the surprising ability of the foetal semi allograft to survive includes alterations in MHC antigens expression and strong secretion of immunoregulatory pregnancy hormones. Logically the mother, successively must balance tolerance to paternal alloantigen’s against a continued necessity for defence against pathogen invasion (Hyde and Schust 2016). Such self-defence is performed through local alterations in decidual immune cell subpopulations combined with alterations in local and systemic immunoregulatory hormones and cytokines secretion (Kutteh et al. 2019). Accordingly, substantial evidence suggests that several immunogenetical and immunological factors may contribute to recurrent miscarriages and reproductive failure in humans. These are as follows: (i) atypical/aberrant HLA expression, (ii) an imbalanced T_h_1 and T_h_2 cytokine network, (iii) corticotrophin-releasing hormone (CRH), (iv) uterine natural killer (uNK) cells expansion and dysfunction, (v) autoimmune diseases, and (vi) roles of innate and adaptive immunity in pregnancy complications. An essential parameter for the M-F immunotolerance has been postulated to be the atypical expression of MHC in human trophoblast. Human trophoblast is known to be a nonrejectable immunological barrier between the mother and the foetus and is characterized by the lack of expression of MHC class II antigens (Makrigiannakis et al. 2011), as well as the lack of expression of classical MHC class I antigens, HLA-A and HLA-B. More specifically, it is well established that the extra villous trophoblast expresses the classical class I product HLA-C and the non-classic MHC molecules HLA-E, -F, and -G (Hunt et al. 2005). The lack of classical MHC class I molecules on the trophoblast prevents allorecognition by maternal T lymphocytes, but poses the problem of susceptibility to MHC non-restricted cytotoxicity systems such as NK cells and lymphokine-activated killer (LAK) cells. Although according to earlier reports the freshly isolated trophoblast is resistant to LAK cell lysis, more rather recent literature suggests that trophoblast may be susceptible to LAK cell-mediated cytotoxicity *via* both the perforin and the Fas/Fas ligand (FasL) apoptotic pathways (Bogović-Crnčić et al. 2007). Binding of FasL to the membrane receptor Fas on specifically activated T lymphocytes for placenta antigens leads to their programmed cell death and, thus would prevent infiltration of maternal lymphocytes into the placenta and reduce their number (Hunt et al. 1997). During implantation and early pregnancy, the immunological processes that take place within the uterus are to a great extent modulated by T_h_1 or pro - (IL-2, IFN γ, and tumour necrosis factor alpha; TNFα) and T_h_2 or anti-inflammatory (IL-4, IL-6, and IL-10) cytokines (Dey et al. 2004). Evidence from studies on human pregnancy points to a strong association between maternal T_h_2-type immunity and successful pregnancy on the one hand and between T_h_1-type immune reactivity and pregnancy loss on the other (Makrigiannakis et al. 2011). An emerging role for the hypothalamic neuropeptide CRH in implantation has been described earlier. The endometrial glands contain high concentrations of CRH, particularly during the secretory phase of the cycle. Oestrogens and glucocorticoids inhibit the promoter of the human CRH gene in transfected human endometrial cells. Progesterone induces the production of CRH in primary cultures of human endometrial stromal cells. In turn, CRH induces stromal decidualization and potentiates the decidualizing effect of progesterone. Additionally, CRH regulates local modulators of the decidualization process. For example, it inhibits the enhancer prostaglandin E_2_ (PGE_2_), induces the inhibitor IL-1, and stimulates the inducer IL- 6 (Makrigiannakis et al. 2011). These modulators exert a positive effect on the synthesis of endometrial CRH, completing this endometrial paracrine network, which could act as a local fine-tuner of decidualization.

The comparisons between human and horse trophoblast cell types, their gene expression, and function make the study of equine pregnancy highly relevant to human health. Indeed, in equine early gestation and the development of the invasive trophoblast of the chorionic girdle and endometrial formation of the gonadotrophin-secreting cup cells appears to represent an atavistic trait more commonly associated with haemochorial placentae of rodents and primates, including humans but not with the more recently derived epitheliochorial placentae of the ungulates (Perissodactyla), including horses (Antczak et al. 2013). The horse has proven to be a distinctively informative species in the study of reproductive immunology for several reasons: (i) unique aspects of the anatomy and physiology of the equine conceptus facilitate approaches that are not possible in other model organisms, such as non-surgical recovery of early stage embryos and isolation of pure trophoblast cell populations, (ii) pregnant mares make strong cytotoxic antibody responses to paternal MHS class I antigens expressed by the chorionic girdle cells, permitting detailed evaluation of the antigenicity of these invasive trophoblasts and how they affect the maternal immune system, and (iii) abundant evidence for local maternal cellular immune responses to the invading trophoblasts in the pregnant mare (Noronha and Antczak 2010).

The relationship between a living organism and its environment is based on a tightly regulated balance between symbiosis and competition. In the viviparous mammals, including humans the immune system generally protects against biological insults, but it may act as friend or foe. It plays a key role in normal reproduction of both sexes. However, it can play a devastating role in infertility. Thus, reproductive immunology is an exciting, ever-growing area of inquiry having applications both in basic reproductive biology/biomedicine and in practical areas such as transplantation immunology/immunogenetics and fertility management by the assisted reproductive technologies (ART).

## 4. Immunogenetics of mammalian reproduction

With the development of immunogenetics and transplantation biology, there came awareness in the 1920s that the conceptus is a uniquely successful natural semialograft. However, it was not until 1953 that focus was really directed on the core immunological phenomenon of pregnancy when Medawar, in his classical review, posed the question, „ How and why does the foetal allograft survive? “Despite rapid advances in general immunology, we cannot yet answer this question satisfactorily.

However, today is well known that the survival of allografts in mammals is influenced by genes of the MHC or MHS. In humans incompatibility with respect to these genes is associated with rapid rejection of foreign tissues (Van Rood and Claas 1990). A remarkable exception is pregnancy, during which (semi)allogeneic foetal tissues avoid rejection. The immunologically privileged nature of the foetal (semi)allograft was first noted by Medawar (1953), and maternal tolerance of an allogeneic foetus is a paradox that remains a central theme in reproductive immunology research today. More recent data indicate that maternal acceptance of the foetal allograft obligatory requires specific recognition of tissue antigens expressed by the genes closely linked to the MHC loci (Ober and Van Der Ven 1997).

The concept of a „major histocompatibility gene “was introduced by Counce and collaborators in 1956 to make a distinction between gene(s) associated with acute rejection of allogeneic tissue and tumour grafts, from those which control chronic rejection of normal allogeneic tissue grafts and usually do not cause rejection of tumour grafts. The latter were called „minor histocompatibility genes “(Counce et al. 1956). Today we know that, in most species, there is not only one gene, but a cluster of genes, and their products are involved in acute (strong) transplantation reactions. Accordingly, this genetic region is called the major histocompatibility gene complex, and represents the most polymorphic and multiallelic genetic system known to date in mammals (Götze 2012).

Our current understanding of the genetics, biology, and physiology of the MHC is primarily based on the results of the fundamental and ingenious research work of Gorrer, Little, and Snell that led to the identification and the thorough genetic analysis of the MHC, termed H-2 in the mouse (Snell and Higgins 1951). Genes of this complex control the expression of histocompatibility allantigens (H-antigens) on cell surface, as well as levels of some serum proteins, were first described in the mouse by Gorer in 1936. His work led to the recognition that the acceptance or rejection of a tumour graft was the direct result of antigenic structures present on the cells and tissue which, if different between recipient and donor, caused the recipient to destroy the graft. He also demonstrated that the relevant antigenic structures, later called H-antigens, can be identified by serological tests (Gorer^1937^). The development of genetically defined inbred strains of mice by Little led to the formal expression of the laws of transplantation: (i) tumour graft exchanged between mice of the same inbred line (syngeneic) are accepted, (ii) tumour grafts exchanged between mice of two different lines (allogeneic) are rejected, and (iii) tumour grafts from parental lines to F_1_ hybrids of two lines are tolerated, but transplants in the opposite direction are not (Little 1941). Further, Snell introduced the concept, and initiated the production of congenic mouse lines. Analyses using congenic mice formed the basis for recent knowledge about the genetics and biology of histocompatibility in general and, in particular, the genetic fine structure of the MHC, its relevance in transplantation biology, immune response, immune cell differentiation, susceptibility to diseases, embryonic/foetal development/survival, and cell biology (Snell 1948). The link-up of the laws of transplantation revealed by the tumour studies with the rejection mechanisms of normal tissue graft suggested by early experiments of Little and collaborators, was extended by Medawar using simple skin-graft techniques. Furthermore, he was able to show that the transplantation reaction represented an immune response by the recipient to the H-gene products (tissue allogeneic antigens) of the donor. Thereby, he and his colleagues Billingham and Brent established the modern approach to allotransplantation of organs and tissues (Medawar 1958). Recognition of these key genes was prerequisite to successful tissue and organ transplantation in humans (Barker and Markmann 2013).

Snell's work in mice led to the discovery of HLA, the major histocompatibility complex, in humans (and all vertebrates) that is analogous to the H-2 complex in mice. Clinical pressure to attempt organ transplantation have led to further intensive efforts for understanding the genetic fine structure, genetic action, regulation and evolution of the MHC not only in the mouse (dominant model for basic immunogenetically oriented research) but also in humans and animal species relevant models for the improvement of clinical transplantation as a therapeutic tool. Now it is recognized that the biological importance of the MHC is not exhibited only in its prominent role in affecting allograft survival, but also in the control of a numerous of biological phenomena, including immune responsiveness, development and susceptibility to diseases, the determination of their own cell surface structures, *i.e.,* CD antigens (which is of key importance for the immune cells), and thereby in cell interactions such as those involved in morphogenesis/ontogenesis as well as in the maintenance of individuality and self-integrity. These discoveries of the MHC implications in the regulation of host defence mechanisms clearly showed its dimension beyond transplantation biology. Briefly, such antigenic products of MHC genes the cell membrane CD antigens apparently serve as self-determinants in both the cellular and the humoral immune responses. The interaction of T cells and self-determinants alone does not lead to their activation/stimulation. However, the concomitant recognition of self and non-self (an infectious agent) induces differentiation to and proliferation of active immune cells of the innate and/or adaptive immunity.

In 1974, Zinkernagel and Doherty demonstrated that the MHC is also involved in the regulation of the cellular cytolytic reaction to virus-infected isogeneic cells or tissue (Zinkernagel and Doherty 1974a). They provided evidence that this control was mediated by H-gene products, but also against isogeneic chemically modified or slightly mutated isogeneic cells with minor H-antigen differences. In general, H-genes appear to be involved in the reactions against cells or tissue which display modified self or genetic traits (Zinkernagel and Doherty 1974b). The transient or continues interaction of the T cells with self-determinants might be considered as the essence of immunological surveillance, and any disturbances of this control/regulatory mechanism may be the basis for the explanation of the predisposition of certain MHC genotypes to susceptibility to infectious and/or autoimmune diseases, including allergic and neoplastic, and even reproductive diseases (Götze 2012).

Their findings, which were published in the journal Nature in 1974, demonstrated conclusively the requirement for the cellular immune system to recognize simultaneously both ‘foreign’ molecules (in the present case from a virus) and self-molecules (in human HLA-antigens). What also became obvious was the important function of the major histocompatibility antigens or HLA in the individual´s normal immune response, and not only in conjunction with transplantation.

Another important finding was the discovery of leukocyte agglutinins by Jean Dausset and collaborators (Dausset et al. 1954), and the attempt to define leukocyte groups using pregnancy sera or post-transfusion sera by Payne (Payne 1957) and van Rood (Van Rood et al. 1958). These studies led to the characterization of the human equivalent of the murine H-2, the HLA system or complex (Götze 2012). The HLA complex contains over 150 gene loci spanning ∼ 4 Mb of DNA on chromosome 6p21. These genes encode proteins controlling cell–cell interactions and regulating immune responses by presenting peptides to T-cells to form immunogenic complexes capable of T-cell stimulation (Toshitani et al. 1996). The MHC is divided into three regions, called the class I, class II and class III (Ober and Van Der Ven 1997). Although the MHC is one of the most extensively studied regions in the human genome, the exact number and function of many of its genes are still not known. However, three classes of genes can be distinguished within the MHC: Class I consists of genes that are involved in the immune responses to: (i) cell-bound antigens, such as virus-infected cells, (ii) chemically modified cells or (iii) otherwise-altered cells, and genes that control the expression of target antigens in the transplantation reaction. These are the so-called histocompatibility H-antigens; Class II comprises genes which are involved in the antibody responses to soluble antigens, so-called Ir genes with their products very likely identical to Ia (Immune response gene associates) antigens and lymphocyte activating determinants, and finally Class III consists of genes that control the expression of certain components of the complement system, particularly those that are involved in the activation of C3-convertase enzyme of the classical pathway and factor B which activates and stabilizes convertases by γ-globulin protein properdin in the alternative pathway (Götze 2012). Moreover, class I and II loci allelic polymorphism is the highest amongst all functional mammalian genes, with some loci exceeding 100 alleles. The class I region is the largest of the three regions, stretching over approximately 2 × 10^6^ base pairs (bp) (Geraghty 1993). Class I HLA molecules are composed of a transmembrane glycoprotein encoded by MHC genes on chromosome 6 and an associated β_2 -_microglobulin encoded by a gene on chromosome 15. The class I genes include the classical or class Ia genes, HLA-A, HLA-B, and HLA-C, but also the nonclassical or class Ib genes, HLA-E, HLA-F, and HLA-G. Class I pseudogenes, such as HLA-H, and gene fragments, such as HLA-J have also been described (Geraghty et al. 1992). The *HLA-E* is expressed in most tissues and in some of the immune cells, including adult and foetal thymus and liver, lymph nodes, spleen, inactivated T and B cells, activated T cells, skin, mucosa of colon, eosinophils, placentas, and extra villous membranes. *HLA-F* transcripts are found in resting peripheral blood T and B lymphocytes, activated T cells, foetal thymus and liver, skin, adult tonsils and lymphoblastoid cell lines (Ober and Van Der Ven 1997). *HLA-G* is expressed in extra villous cytotrophoblasts at the M-F interface (Kovats et al. 1990; Yelavarthi et al. 1991) and in the anterior chamber of the eye and foetal thymus, first trimester foetal liver (Houlihan et al. 1992). Low levels of HLA-G expression were detected in adult peripheral B and T lymphocytes (Kirszenbaum et al. 1994), mononuclear phagocytes (Yang et al. 1996) and progenitor haematopoietic stem cells (CD34^+^) from umbilical cord blood (Kirszenbaum et al. 1995). For a detailed review of the class I region genes see Geraghty (1993). Class II molecules consist of two transmembrane glycoproteins (cx and fI chains), both encoded by MHC genes. Class II gene products have more limited tissue distributions than class I antigens, being restricted primarily to B lymphocytes, macrophages, endothelial cells, and activated T cells. Additional non-HLA class II region genes have been identified by molecular genetic techniques, including genes encoding antigen-processing and transport proteins, such as: *TAP 1*, formerly *RING4, TAP2*, formerly *RINGII, LMP7*, formerly *RINGJO*, and *LMP2*, formerly *RINGJ2* (Trowsdale et al. 1990). The class III region contains genes encoding complement components (C4A, C4B, C2 and BF), the cx and fI chains of the tumour necrosis factor, the enzyme cytochrome P450 steroid 21-hydroxylase, and the heat shock protein Hsp70. Additional genes have been mapped to this region but have not yet been characterized. Molecules derived from the HLA-D region are called class II antigens. They are heterodimeric glycoproteins expressed on the surface of B lymphocytes, macrophages, epidermal Langerhans cells, and, under certain circumstances such as the M-F interactions (Gerenčer and Kaštelan 1983), on activated T cells and some epithelial cells. The most if not all class II genes appear to be localized to three well-defined loci called DP, DQ, and DR (Ober et al. 1985). Paradigms from transplantation immunology have provided models for investigating the M-F HLA compatibility on pregnancy outcome and the development of treatments for the prevention of foetal loss (Beer et al. 1981; Ober and Van Der Ven 1997).

However, significant differences between foetal (semi) allografts and tissue allografts have come to focus almost at the same time, particularly with discovery of the absence of classical HLA antigens (although the class Ia genes HLA-A, HLA-B and HLA-C have a ubiquitous tissue distribution and are expressed on nearly all nucleated cells) on foetal tissues that are in contact with maternal tissues during pregnancy (Johnson and Stern 1986). Instead, a nonclassical HLA gene, HLA-G is expressed at the M-F interface (Kovats et al. 1990; Yelavarthi et al. 1991). The HLA-G has many unique properties in addition to its tissue distribution, which may effectively modulate maternal immunological tolerance during pregnancy (Schmidt and Orr 1993) as the HLA-G is expressed on syncytiotrophoblasts and cytotrophoblasts, which lack expression of HLA-A and HLA-B (Kovats et al. 1990; Schmidt and Orr 1993).

However, the function of a relatively novel and unique HLA-G gene is still largely unknown. Due to their genetic polymorphism, the number of different class II antigens expressed by a single cell varies depending on whether the cell is homozygous or heterozygous in regard of the HLA-D region (Gerenčer and Kaštelan 1983). All class II molecules are composed of one *a* and one *f3* chain. The HLA-D region contains multiple genes encoding such polypeptide chains. The *a* and *f3* chains have very similar structures and are obviously evolutionarily related (Götze 2012). Even their gross configurations suggest that the ancestral class II molecule was a homodimer. Therefore, it seems reasonable to conclude that *a* and *f3* chains diverged during evolution by processes involving gene duplication and specialization. Following the emergence of genes encoding heterodimers, further duplication events gave rise to an array of genes similar to those coding for the original *a* and *f3* chains. By means of a number of techniques it has been possible to demonstrate that such genes occur to a large extent as clusters in the HLA-D region (Gerenčer and Kaštelan 1983).

The survival of allografts in mammals is influenced by genes of the MHS. In animals and humans incompatibility with respect to MHS genes is associated with rapid rejection of foreign tissues. A notable exception is pregnancy, during which semiallogeneic foetus escape rejection. Paradigms from transplantation immunology have provided models for investigating the M-F relationship, both with respect to the influence of M-F HLA compatibility on pregnancy outcome and the development of treatments for the prevention of fetal loss.

## 5. MHC modulates foetal-maternal tolerance, transplantation reactions and controls immune responses in mammals

The MHC is a genetic region that has been intensively studied for almost the past 6 decades. Interest in the MHC has been high because of: (i) the particular involvement of the MHC in transplantation reactions, including organ allograft rejection in humans and foetal semiallograft tolerance or rejection in all mammals, and (ii) the more general role of MHC gene products in the genetic control of immune responses in mammals. The MHC has several remarkable properties that include a distinctive genetic structure which has been well-preserved through evolution, and the extreme plasticity or polymorphism of form of the principal MHC genes, which can coexist within a single species in 30 or more allelic forms. The genes of the MHC regulate cell-cell interactions of various types within the lymphoreticular system, and thus function as the so-called "immune response" genes that have been described in laboratory rodent models. In humans, the "disease associations" demonstrated between MHC alleles and various pathologic conditions are probably manifestations of abnormal functions of immune regulation governed by the MHC. Although, studies of the MHC have been performed in swine, cattle, horses, sheep, goats, dogs, cats and chickens, data obtained regarding the MHC in domestic animal species are still insufficient. This paragraph is summarizing the current knowledge about reproductive immunology in different species of prevalently domesticated viviparous mammals, and is based on the anticipation that in the next few years studies on the MHS, immune response (Ir) genes and their products in veterinary relevant species will be intensified due to the fact that the association between tissue antigens, Ir genes and specific immune responsiveness may offer valuable tools for animal selective breeding, particularly in food animals in order to improve health programs and, thus their productive and reproductive efficiency (Van Dam 1981).

However, the MHC has been given different names in different species. It is designated H-2 in the mouse, HLA in humans, B in the domestic fowl, RT1 in the rat, and Smh in the mole rat. In most of the other species that have been studied, the MHC is referred to by the LA symbol (for lymphocyte or leukocyte antigens), prefixed by an abbreviation of the species common name (Klein 1986). Thus, it is called ChLA in the chimpanzee, GoLA in the gorilla, RhLA in the rhesus macaque, RLA in the rabbit, BoLA in the domestic cattle, SLA in the swine, and so on (Table 1).

**Table 1.** Terminology used for the major histocompatibility complex (MHC)/major histocompatibility system (MHS) of humans and various animal species.

Species	Term for MHC/MHS	Reference*
Human	HLA	Amos 1968
Chimpanzee	ChLA	Balner et al. 1978
Rhesus monkey	RhLA	Balner et al. 1971
Mouse	H-2	Klein 1979
Rat	RT1	Gȕnther and Stark 1979
Guinea pig	GPLA	Geczy et al. 1975
Syrian hamster	Hm-1	Duncan et al. 1977
Rabbit	RLA	Tissot and Cohen 1972
Chicken	B	Hȕla 1977
Swine	SLA	Vaiman et al. 1970
Bovine	BoLA	Caldwell 1979
Sheep	ShLA/OLA	Millot 1978
Goat	GLA	Van Dam et al. 1979
Horse	ELA	Lazary 1980
Dog	DLA	Dausset et al. 1971
Cat	FLA	Yuhki and O'Brien 1988

* By the authors that recognized, characterized and described novel or particular information/knowledge about tissue antigens.

This practice has two problems associated with it. The first, is that MHC products are expressed on many other tissues in addition to lymphocytes or leukocytes and lymphocytes express many other antigens (as CD antigens) in addition to those controlled by the MHC, and their antigenicity is secondary to their biological function in defence of an organism, such as humans where their MHC is termed HLA. Secondly, the use of common names to identify a species is a potential source of confusion, because common names are notoriously vague and imprecise. Obviously, common names not only fail to identify the species appropriately, they often do not even identify the genes or the family. If the trend in choosing common names for MHC symbols were to continue, chaos would soon ensue because we can expect that MHC in many different species to be identified in the future (Klein and Schönbach 1993). In particular, further research on the MHC of domestic animals is of great importance, both for its contribution to the overall understanding of the biological significance of the MHC and for its practical application in clinical veterinary and human medicine, but also in clinical transplantation in biomedicine, in general (Antczak 1982).

The placenta is essentially the M-F interface, functioning as an anatomical and histological barrier between foetus and mother with separate circulatory systems. However, the placenta is populated with immune and accessory, antigen presenting cells (APC), including uterine uNK cells (70%), macrophages (20%), T cells (10%), including CD4^+^, CD8^+^, γδ T cells, regulatory T cells, and scarce dendritic cells and B cells. The numbers of these cells and roles that they play differs throughout the various stages of pregnancy. Today are well recognized several local and systemic immuno-endocrine and molecular modifications that are proposed to be involved in protection of the developing foetus from rejection by the maternal immune system (Kutteh et al. 2019). These mainly include: (i) cytokine balance shift, which is in the successful pregnancy associated with a predominance of T_h_2-type immunity, and induction of T_h_1-type responses only if considered potentially dangerous for the continuation of pregnancy. This paradigm has been expanded to consider T_regs_ and T_h_17 cells, with T_regs_ playing pregnancy-protective roles and T_h_17 responses being detrimental to pregnancy, (ii) influence of female sex hormones, in a way that the oestrogen and progesterone levels are massively upregulated during pregnancy, and that both hormones have been shown to have immunomodulatory functions, impacting immune cell recruitment, distribution and reactions which may be detrimental for foetal survival, and (iii) unique HLA gene expression by trophoblasts, the main cell type of the placenta which exhibit particularly different MHC tissue antigens comparing those of mother and of foetus. In this regard, the MHC class II molecules are not expressed by trophoblasts while of the class I molecules such as HLA-C, E, F, and G are expressed by trophoblasts and have tolerogenic functions including control of depth of trophoblast invasion and binding to inhibitory NK cell receptors (Hunt et al. 2005). On the other hand, such aberrant expression of MHC tissue antigens on trophoblast increases its susceptibility to lysis by MHC non-restricted NK and LAK cells either *via* perforin or FasL - induced apoptotic pathways (Bogović-Crnčić et al. 2005).

A shift in the balance of T_h_1/T_h_ 2 cytokine production by maternal peripheral blood leukocytes (PBL) is regarded as a common important feature of successful mammalian pregnancy. Although the phenomenon has been studied extensively in mammals with invasive haemochorial placentae such as higher order primates (like apes and monkeys), including humans, and lagomorphs/rodents (like rabbits/guinea pigs, mice and rats), the paradigm has not been studied in detail in species with less-invasive either epitheliochorial (swine, horses and lower order primates) or synepitheliochorial placentae such as ruminants (like cattle, sheep and goats), respectively (Chavatte-Palmer and Tarrade 2016). Several earlier studies mostly in sheep have clearly documented that there were no detectable differences in antigen-driven PBL proliferation, IFN γ, IL-4 or IL-10 production between pregnant and non-pregnant sheep. These data suggest that a shift in T_h_1/T_h_2 cytokine production does not occur in pregnant sheep and indicate that further comparative reproductive immunology studies on species with non-invasive placentation will be useful for further understanding of the M-F interactions and immune regulation during pregnancy (Wattegedera et al. 2008). There are also animal studies demonstrating that prostaglandins (PG) inhibits the expression of IL-17 in a dose-dependent fashion in pregnant cows (Maeda et al. 2013). Interesting findings were reported by Cheng et al. (2016) who evaluated plasma PG concentrations and expressions of three T_h_ - related cytokines, T_h_ 1 (IL-1β and IL-6) and T_h_ 2 (IL-4,) in pregnant cows during early pregnancy following artificial insemination (AI) and in non-pregnant cows. None of the cytokines was associated with a change in plasma PG, which differs from what occurs in mice and humans during early pregnancy. In the pregnant rat, PG and dexamethasone markedly inhibited the expression of IL-6 in the *corpus luteum* throughout normal pregnancy, which might have a deleterious effect on luteal function (Telleria et al. 1998). Furthermore, the inhibited IL-6 expression will in turn reduce the progression of naïve T cells to T_h_17 cells, and numerous studies have shown that optimal IL-6 regulation may be crucial to the avoidance of pregnancy morbidities and to carrying successful pregnancies to term. Accordingly, the expression patterns of the IL-6 family of cytokines may have the potential as new biomarkers for the treatment of reproductive pathologies in order to avoid fetal rejection (Markert et al. 2011).

A notable exception is pregnancy, during which semiallogeneic foetal tissues avoid rejection. The immunologically privileged nature of the foetal semi allograft was first observed by Medawar in 1953 (Medawar 1958), and maternal acquired immunological tolerance of an allogeneic foetus is a paradox that remains a central theme in reproductive immunological research today (Makrigiannakis et al. 2011). Indeed, an immunological paradox is presented by the state of human pregnancy, in which genetically different tissues reside side by side in apparent harmony. This has given rise to the metaphor of transplantation immunology and allograft either rejection or tolerance. In 1953, Medawar suggested mechanisms that could account for the surprising ability of the foetal semi allograft to survive in a potentially hostile environment. Although somewhat different from Medawar’s original ideas, mechanisms of foetomaternal immunotolerance are now thought to involve the spatiotemporally coordinated effects of numerous hormonal and immunological factors that modify the maternal immune system’s response to the semi allogeneic conceptus. Since then, much research has been performed to investigate why the foetus in most pregnancies, in spite of being semiallogenic, is not rejected by the immune system. Experiments in transgenic mice have suggested that dysfunctions in both the innate immune system (NK cells) and the adaptive immune system (T-cells and T regulatory cells) result in increased foetal loss rate **(**Christiansen 2013).

Many studies have suggested that women with pathological pregnancies such as recurrent miscarriages have signs of generally exaggerated inflammatory immune responses both before and during pregnancy and signs of breakage of tolerance to autoantigens and foetal antigens. In addition, several abnormalities of innate immune responses seem to characterize women with pathological pregnancies. These abnormalities involve disadvantageous interactions between uNK cells and HLA-G and HLA-C on the trophoblast that may have pro-inflammatory effects. Also, humoral factors belonging to the non-pregnancy specific biomarkers of innate immune system such as mannose-binding lectin (MBL) seem to be associated with pregnancy outcome probably by modifying the level of inflammation at the M-F interface. The pro-inflammatory conditions at the M-F interface characterizing pathological pregnancy are suggested to predispose to adaptive immunological processes against alloantigen’s on the trophoblast that may further increase the risk of pathological pregnancy outcome (Laškarin et al. 2005). After binding to mannose-rich oligosaccharides on the surface of microbes, the lectin pathway of the complement system is activated. Furthermore, MBL acts as an opsonin enhancing the phagocytosis of the microbes by macrophages, and also may participate in clearing of apoptotic cells, cell debris (particularly of importance at the M-F interface surface), and thus, supports maintenance of the anti-inflammatory conditions (Heitzeneder et al. 2012). The best documented adaptive immune reaction against foetal alloantigen’s is directed against male-specific minor histocompatibility (HY) antigens expressed on male foetal and trophoblast cells. Anti-HY immunity seems to play a role especially in women with secondary recurrent miscarriage **(**Christiansen 2013).

It is now recognized that the biological importance of the MHS lies not only in its pre-eminent role in affecting allograft survival, but also in the control of a large array of biological phenomena, including immune responsiveness, development and susceptibility to diseases in animals and humans. However, in animals which are important in livestock production, as well as in recreation animals, the associations between tissue antigens and specific immune responsiveness could offer valuable tools for breeding and health programs. Thus far, parts of the MHS of chicken, dog, swine, horse, cattle, sheep and goat have been characterized. However, with the exception of chicken, the information on Ir gene expression and association of tissue antigens with diseases is very limited yet.

## 6. Reproductive and transplantation immunology: current status

With development of immunogenetics and transplantation biology the attention of scientists in human medicine was focused on the central immunological paradox of foetal survival as semi allograft (Billingham and Beer 1984). Knowledge of the immunological/immunogenetical mechanisms involved could lead to improved treatment of patients with habitual abortions or primary infertility, in specific contraceptive therapy, and in the management of patients with foreign tissue transplants or tumours as well as for early predictions/preventions of genetically inherited autoimmune diseases, and increased genetic susceptibility to infectious diseases (Toshitani et al. 1996; Blackwell et al. 2009.).

### 6.1. Immunology of reproduction and transplantation in humans

Human reproduction is remarkably inefficient compared with that of other mammalian species. Approximately 70% of spontaneous conceptions are lost prior to completion of the first trimester. Implantation failure and pre-clinical losses account for 85% of total pregnancy losses, and clinical miscarriage for 15%; yet these percentages may underestimate the actual frequency of reproductive failure (Makrigiannakis et al. 2011). Humans are also relatively infertile species and recurrent pregnancy loss (RPL) also called recurrent spontaneous abortion (RSA) in either specific women or couples is common but incompletely understood problem. Such miscarriages were defined as 3 or more consecutive RPL prior to week 20 of gestation and the immune mechanisms were often postulated as responsible when other explanations have been disapproved. Thus, it has been reported based on a 5-year human clinical study designed to address whether naturally acquired microchimerism, which a daughter acquired from her mother could be detected in unrelated women with RSA, that differences in microchimerism versus control normal pregnancies were indicative (Croy 2014b). Earlier studies into immune functioning during pregnancy in mammals, including humans hypothesized pregnancy as a time of immune suppression, when maternal immune responses were presumably down-regulated in order to avoid rejection of the foetus (Chen et al. 2012). It is well known that immune function is the result of a highly complex system of cellular and molecular components, working in intricate parallel and competing pathways to defend the host from invasive pathogens and destroy harmful or senescent/damaged cells of the host, while avoiding production of potentially harmful chronic inflammatory factors. These systems must adapt to the prolonged exposure of the semiallogeneic embryo, in ways not yet fully understood. Furthermore, cells and molecules evolved to function in the immune system have, in mammals, been evolutionarily adapted for reproductive functions, comprising critical components of fertilization, embryo implantation, pregnancy health, and parturition. It is remarkable that immune system genes have been identified as among the most recruited into endometrial expression during the evolution of placentation (Lynch et al. 2015). Thus, it is not surprisingly that immune system dysfunction has been recognized as a likely contributor to endometriosis, infertility, RPL, preeclampsia (PE), and preterm delivery. From the perspective of cellular immunity it is well known that T lymphocytes play a crucial role in adaptive immunity, and that shifts among T cell subsets occur during normal pregnancy. The initial research pointed to a shift from CD4^+^ T helper cells type 1 (T_h_ 1; pro-inflammatory) towards type 2 (T_h_ 2; anti-inflammatory) (Larocca et al. 2008). This simplistic paradigm has been proven to be insufficient, as inflammatory antimicrobial morbidities and autoimmune conditions appear to change throughout pregnancy, implying a need for more precise analysis of the kinetics of immune cells and their responses during pregnancy. Such a cell subset is T_h_17 cells, a novel lineage of CD4^+^ T helper cells, which can be both pro- and anti-inflammatory within different tissue sites, including placenta and reproductive tract in pregnancy. These cells were found to be located within an appropriate niche in decidual tissue for correct interactions with regulatory T_reg_ cells and showed the spectrum of outcomes from inappropriate T_h_17 regulation varies with the PE being a less severe disturbance than pregnancy loss (Croy 2014b). T_h_17 cells have great importance in host defence against extracellular infectious microbes by amplifying neutrophilic responses, promote B-cell subset switching, activate mucosal barrier of epithelial cells and produce antimicrobial peptides. Also, these cells are involved in the pathogenesis of some autoimmune conditions and have a rapid response at the site of inflammation, and thus, participate in physiological pregnancy as well as in the pathogenesis of pregnancy disorders, such as RPL and PE. In contrast, T_reg_ cells maintain tolerance by inhibiting either the production of cytokines and immunoglobulins or the proliferation of NK cells and dendritic cells (DC), respectively (Laškarin et al. 2007; Saito et al. 2010). Naive forms of helper (CD4^+^) and cytotoxic (CD8^+^) T cells are considered immature and, unlike activated or memory T cells, has not encountered its cognate antigen within the periphery, and are prompted to differentiate into T_h_17 in response to interleukin (IL) – 6 and transforming growth factor (TGF) – β, secreted primarily by innate immune cells. T_h_17 cells are developing in the presence of IL-23, and mature T_h_17 cells produce several cytokines, such as IL-17 (A or F) and IL-22. When IL-17 stimulates innate immune cells to increase their secretion of IL-6, and when TGF-β is present in the absence of IL-6, regulatory T_reg_ cells will develop instead of T_h_17 cells (Osborne et al. 2019). The balance between these two types of CD4^+^ T helper cells, one promoting inflammation (T_h_17) and the other (T_reg_) controlling adaptive immunity is strictly regulated by the presence or absence of IL-6 during the course of pregnancy in order to prevent inflammatory morbidities, and autoimmune diseases to avoid foetal rejection (Saito et al. 2011).

While our understanding of the role of T_h_17 and T_reg_ cells in physiological pregnancy is still improving, the balance between immune defence and immune tolerance is crucial for maintaining a normal pregnancy and attaining its healthy outcome. However, that balance is changing over the course of pregnancy, with T_reg_ cells higher in early pregnancy to support tolerance of the foetus, but lower in late gestation than their counterparts T_h_17 cells. The later cells are essential for immunological defence of the host against pregnancy morbidities commonly inducing infections at the M-F interface and for promotion the initiation of parturition by producing higher amounts of pro-inflammatory molecular (IL-6 and IL-17) and cellular elements (γδ T cells, NK cells) of the innate immunity, but without deleterious effects on foetal-maternal tolerance (Figueiredo and Schumacher 2016). Animal studies indicate that adequate frequencies of T_reg_ cells are essential at the maternal-foetal interface for immunological tolerance at implantation (Saito et al. 2010). However, this dominance may be a relative increase only, as T_h_17 cells are in higher numbers in the decidua than they are in the periphery (Wu et al. 2014) due to the fact that they are necessary for immunological defence against infections at the M-F interface, with numbers that may be increased in pregnancy as compared to the non-pregnant status. Also, it may be that the type of T_h_17 cells at the M-F interface is different from that found in the periphery, with more γδT cells of a type that produces higher amounts of IL-17. These data suggest a pregnancy specific recruitment or distribution of IL-17-producing cells at the M-F interface, with even higher numbers in allogeneic as opposed to syngeneic pregnancies (Pinget et al. 2016). T_h_17 products, prevalently IL-17 in the human placentae from both normal pregnancy outcomes and spontaneous abortions play a role in angiogenesis and immune regulation, which is not exhibited in murine placentae and, thus current mouse models may not be useful in describing these roles (Pongcharoen et al. 2007). Whether and how T_h_17 cells at the maternofoetal interface are active in pregnancy (and at what times) depends not only on the presence of these cells and their regulatory counterparts T_reg_, but also on the actions of hormones and signalling pathways that change during pregnancy. The discovery of the role of the MHC or MHS genes/cell surface antigens in the regulation of host defence mechanisms such as those involved in morphogenesis and the maintenance of individuality and self-integrity, gave the MHC a further dimension beyond transplantation biology. Namely, the clinical transplantation is often complicated by rejection episodes, in which the immune system of the recipient reacts to the foreign transplantation MHC antigens on the graft. This immune response includes humoral and cellular components. In the first phase, B lymphocytes form antibodies to the MHC alloantigens. In the second phase, CD8^+^ T lymphocytes recognize and react to MHC class I antigens, and CD4^+^ T cells react to MHC class II antigens. The frequency and severity of these rejection episodes can be diminished by immunosuppressive drugs, MHC matching between donor and recipient, and immune modulation by blood transfusion. Insight into the factors that influence the T and B cell repertoire after blood transfusion might lead to new approaches to improve graft survival (Van Rood and Claas 1990). Otherwise, the survival of allograft in mammals, including humans is influenced by genes of the MHC associated with rapid rejection of foreign tissues (Ratner et al. 1991).

The RPL of unknown aetiology like the RSA may be more a syndrome than a single aetiology disease. Many studies in humans have shown that couples with RSA of unknown aetiology had significantly higher HLA compatibility than couples with normal pregnancies (Gerenčer et al. 1988; Ho et al. 1990). However, elucidation of the basic mechanism which plays the major role in RSA of unknown aetiology is necessary to understand the role of HLA compatibility in this type of a reproductive failure (Christiansen 2013). During embryonic development, immune rejection provoked by semiallogeneic antigens of the foetus is inhibited by the mother (Chen et al. 2012), and the immune balance between Th1 and Th2 cells was thought to play a key role in the embryo implantation (Larocca et al. 2008; Saito et al. 2010). On the other hand, the Th17 and T_reg_ T cell balance has become increasingly important in reproductive immunity research (Osborne et al. 2019). Th17 cells that mediate immune rejection and Treg cells that mediate immune tolerance maintain a dynamic balance and a normal immune status of the organism (Saito et al. 2011). Changes in peripheral blood immune cells reflect the systemic immune status and the decidual NK cells reflect the local immune status of the M-F interface microenvironment (Wu et al. 2014; Figueiredo and Schumacher 2016). After embryo implantation, the endometrium serves as the primary and earliest tissue constituting the M-F interface microenvironment, and the recruitment of Treg cells play an important role in local immunity, *i.e.,* in immune rejection or tolerance by the mother caused by semiallogeneic antigens of the foetus (Makrigiannakis et al. 2011). Therefore, simultaneous detection of Th17 and T_reg_ cells in peripheral blood and decidual tissue may help to understand the immunological pathogenesis of the RSA (Osborne et al. 2019). Accordingly, possible immunological pathogenesis involved in the RSA of unknown aetiology may be associated with insufficient recruitment of T_reg_ cells in endometrial tissue and the Th17 and T_reg_ cell imbalance may be an important immune factor in the pregnancy failure. The alternative explanation of the RSA of unknown aetiology is that it has a genetic background which is based on the large number of observations showing the presence of lethal and semi lethal genes in the mammalian genome (Götze 2012). Studies in humans have shown that the increased HLA sharing in the RSA couples can occur at all loci, but sharing at HLA-D/DR locus appears at higher frequency (Gerenčer et al. 1983). Such observations that there is significantly increased HLA sharing in couples with the RSA (Gerenčer et al. 1979, 1988; Ho et al. 1990) were interpreted by existence of HLA-linked recessive lethal genes or genes responsible for normal growth and development of the foetus. The strongest correlation between HLA sharing and the RSA was found when sharing occurred at three or more HLA loci (HLA haplotype sharing) such as: HLA-A, -B, -D/DR and -DQ (Ho et al. 1990). Also, the HLA-G locus, a MHC class I antigen encoded by a gene on chromosome 6p21, plays an important role in the RSA (Hunt et al. 2005). The HLA-G differs from classical HLA class I molecules by restricted tissue distribution and limited polymorphism, and its role in immune tolerance was not fully defined by studying HLA-G expression in trophoblastic cells (Kovats et al. 1990). Several studies have found an aberrant or reduced expression of HLA-G protein in pathological conditions such as preeclampsia or RSA (Yelavarthi et al. 1991) in comparison with normal placentas. This finding strongly suggests that HLA sharing is only a marker for HLA-linked gene(s) (Kirszenbaum et al. 1994, 1995; Yang et al. 1996) that are involved in the pathogenesis of the RSA. The fact that women who have the RSA with one partner often do not have the RSA with another partner (Schmidt and Orr 1993) supports this hypothesis. Unfortunately, if the mechanism of the RSA in humans is dominantly genetic, the treatment at the present biotechnological and scientific level of knowledge as well as ethical principles in biomedicine is not possible.

An excellent overview (Bogović-Crnčić et al. 2005) of the earlier findings on the complex immuno-endocrine interactions that occur at the M-F interface is offering a scientifically based explanation of the immunological enigma of maternal tolerance of the foetal semi allograft, particularly during early pregnancy. The massive presence of uNK cells and cytolytic mediators PER and FasL at the M-F interface raises a question of their role(s) in the immunological relationship between maternal tissues and trophoblast cells in normal pregnancy. Thus, it is very likely that hormonal and Th1/Th2 cytokine balance and production dynamics modulate uNK cells activities towards immunologic tolerance of the foetus and maintenance of pregnancy (Bogović-Crnčić et al. 2005).

Another interesting review focuses on the characterization of decidual macrophages (DM) and DC, and their involvement in cell-cell interactions within the DL network, which are likely to influence uterine and placental homeostasis and maternal immune responses to the foetus during pregnancy. A delicate balance of innate and adaptive immune responses at the M-F interface promotes survival of the semiallogeneic embryo and, at the same time, provides effective immune responses to protect the mother from environmental pathogens. Maternal APC, DM and DC scattered within decidualized endometrium perform antigen handling and processing, a primary event in the onset of immune responses which may determine their stimulatory or tolerogenic nature (Laškarin et al. 2007).

Current understanding of DC immunobiology within the context of mammalian M-F tolerance was reviewed and discussed herein. Foetal and maternal immune cells come into direct contact at the decidua, a highly specialized mucous membrane that plays a key role in foetal tolerance. Uterine DC within the decidua has been implicated in pregnancy maintenance by serving as the APC, with the unique ability to induce primary immune responses. The identified DC subsets have been shown that differentially control lymphocyte function namely, the DC may also act to induce immunologic tolerance and regulation of T cell-mediated immunity (Blois et al. 2007).

The tumour-associated glycoprotein-72 (TAG-72) is physiologically present in secretory phase endometrium, but its presence and possible immunological role in early normal human pregnancy decidua has not received much attention. In the last decade much research on its putative beneficial effect for the maintenance and success of pregnancy have been performed by many authors, including Rukavina and his colleagues. They obtained rather novel and detailed data emphasizing the anti-inflammatory properties of TAG-72-treated decidual CD1a^+^ DC cells in terms of their interaction with T cells. Thus, the absence of TAG-72 at the M-F interface during early pregnancy could lead to a mild pro-inflammatory response that may be beneficial for pregnancy success and trophoblast growth control (Laškarin et al. 2011).

The mechanisms of the M-F immunotolerance in mammals involve the spatiotemporally coordinated effects of numerous hormonal and immunological factors that modify the response of the maternal immune system to the semiallogeneic conceptus. These factors primarily include PG, Th1/Th2 cytokines, cytolytic cells (NK and T cells), cytolytic molecules (PER and granulysin; GNLY), secretory molecules (mucins, MUC1 and TAG-72) and APC, prevalently DC. Communication between the genomic and non-genomic PG-regulated signalling pathways could be of critical importance for the establishment of optimal endocrine-immune interactions in the human endometrium during the initiation and maintenance of pregnancy (Veljković-Vujaklija et al. 2011b).

The GNLY is especially abundant in the NK cells which are able to spontaneously secrete high quantities of this cytolytic molecule. Besides being a potent bactericidal and tumouricidal molecule, the GNLY is also found to be a chemoattractant and a proinflammatory molecule. The precise role(s) of the GNLY at the M-F interface has not been elucidated as yet. According to several authors it is possible that GNLY plays a double role by acting as an immunomodulatory and a host defence molecule by protecting both the mother and the foetus from a wide spectrum of pathogens. Simultaneously, on the other hand, in case of the NK cells activation, it may act as an effector cytolytic molecule by causing the apoptosis of semi allograft trophoblast cells, which consequently lead to various pregnancy disorders or pregnancy loss (Veljković-Vujaklija et al. 2012).

During gestation, many different mechanisms act to render the maternal immune system tolerant to semiallogeneic trophoblast cells of foetal origin, including those mediated *via* mucins that are expressed during the peri-implantation period in the uterus. The TAG-72 enhances the already established tolerogenic features of decidual DC with the inability to progress towards Th1 immune orientation due to lowered IFN-γ and IL-15 expression. Muc 1 supports activation of decidual macrophages, restricts the proliferation of decidual regulatory CD56^+^ oNK cells, and downregulates their cytotoxic potential. Removing TAG-72 and Muc 1 from the eutopic implantation site may contribute to better control of trophoblast invasion by NK and T cells and appears to have immunologic significance for normal implantation. However, these processes may lead to uncontrolled trophoblast growth after implantation, inefficient defence against infection or tumours, and elimination of immune cells from the M-F interface (Redžović et al. 2013).

Although, it is still not elucidated how the feto-placental unit or the semiallogeneic foetal graft in most instances evades rejection by the maternal immune system, it has become more and more clear, that the M-F interface is not an immunologically inert area. On the contrary, the obtained results showed that a series of active immune and endocrine processes, some of which have specificity for paternal foetal antigens, take place at the maternal side of the trophoblast during pregnancy. It is noteworthy to mention that immunologic implications of pregnancy in viviparous mammals, including humans are hormonally regulated and directed on molecular and cellular components of the mucosal immune system located in uterine decidua and a unique immunological environment of the M-F interface. Logically, the research focus has been oriented towards these sites, and resulted in important findings as previously described.

Biomedical animal research is almost totally a murine affair. Undoubtedly, the laboratory mouse has proven to be an invaluable model for biomedical research and most of what we know today about mammalian basic and applied biology is obtained from research performed on rodents and rabbits. Nonetheless, to neglect other animal models certainly is to ignore the need to address evolutionary divergence among mammals by studying biology across an array of phylogenetically related genotypes. Moreover, the opportunity to exploit unique biological models or intriguing insights is highlighted whereby farm animals are being used to develop concepts pertinent to a wide range of mammalian species, including humans (Hansen 2010). An increase in interest in basic biomedical research using farm animals as Medawar capitalized on the unique properties of the placental vasculature of twin calves to recognize clarity about the nature of immunologic tolerance (Medawar 1961). Such interest will have a positive impact not only on cattle productivity but as well on understanding mammalian biology, in particular reproductive and hence transplantation immunology and thus, improving human health. Generally, it has become evident that advances in farm animal reproduction have become increasingly dependent on improved understanding of the immunology of maternal reproductive tract responses to sperm cells and seminal fluid that are antigenic for females. The immunization to these male antigens has potential consequences for fertility and pregnancy outcome (Vaiman et al. 1978; Tripathi et al. 1999). Deposition of semen in the female reproductive tract leads to an inflammatory response to semen, and contributes to the removal of sperm by the innate immune response (Schuberth et al. 2008) and thereby prevents the adaptive immune response against allogeneic antigens of sperm cells.

### 6.2. Immunology of reproduction in mammals of veterinary relevance

Immunology of reproduction attracted much more attention for research in humans and laboratory animals than in farm or companion animals and horses, but its significance is also of outstanding interest in veterinary medicine as well, particularly in veterinary reproductive immunology of viviparous mammals. Immunological paradox, that is known for a long time, is a physiological either tolerance or activation of the immune response to seminal and sperm antigens in both females and males. Indeed, the sperm cells are foreign even to males because they appear late in puberty so the immunotolerance to sperm antigens is not established. However, in testes spermatozoa are protected by blood-testis barrier preventing contact with the immune cells of the male (Johnson and Setchell 1968). If this barrier is somehow damaged (by extremely low environmental temperature, infection or trauma), an autoimmune reaction occurs, and by using this paradigm, as early as in 1951, Voisin and collaborators developed a model of experimental autoimmune orchitis. Besides, submucosa of the male reproductive tract is heavily populated with the suppressor T cells preventing initiation of an immune response to autologous spermatozoa despite their isoantigenic properties (El Demiry and James 1988). Ever since the initial insights, it was thought that seminal plasma (SP) in mammals serves only to provide adequate ejaculate volume, to deliver energy (fructose), to maintain optimal pH and to protect sperm cells from oxidative damage. However, the SP properties are shown to be of extreme importance due to its multifunctional potentials not fully recognized as yet. Interestingly, the molecules that are usually found in other body fluids are also present in very low concentrations in the SP only. These are: citric acid, alkaline phosphatase, phosphoryl-choline, prostaglandins, fructose, ergothioneine, inositol and glyceryl-phosphoryl-choline. Also, its composition may significantly vary depending on animal species, age, season and T serum level (Mann 1974). Therefore, the SP is an extraordinary complex body fluid in the molecular sense, and thus one of the most confusing questions in this area of reproductive immunology was: why is something so complex like the SP if there are only few of its roles? Excellent reviews were authored by James and Hargreave (1984) and Witkin (1988) offering, at least partly, an explanation of this paradox. In the late 1970s it was discovered by the *in vitro* studies that constituents of SP may inhibit functions of the immune system cells, thus preventing recognition of spermatozoa as foreign cells in the female genital tract. Bovine cervical mucus (CM) also exerts strong immunosuppressive effects (Matoušek et al. 1988) as well as bovine and horse ovarian follicular fluids (Matoušek et al. 1985). Stites and Erickson (1975) were the first who reported the immunosuppressive effects of the human SP towards lymphocyte activation and responses of female or male to allogeneic or autologous sperm cells, respectively. This finding was confirmed later by others (Franken and Slabber 1981), and attracted great scientific interest because such immunosuppressive factors may also contribute to the development of sexually transmitted diseases, such as the acquired immunodeficiency syndrome (AIDS), and to the growth of urogenital malignancies, including carcinoma of the cervix in humans (James and Hargreave 1984). In 1975, Prakash et al. (1976) noted the same effect of human SP on mouse lymphocytes. Lugaro et al. (1984) discovered a low-molecular weight factor in bovine SP that inhibits RNA synthesis. Further information regarding immunosuppressive effect of bovine SP were provided by Fahmi et al. (1985a,b). Moreover, Fahmi and Hunter (1986) found two active constituents in bovine SP, one of a high and one of a low molecular weight. Following separation of the bovine SP, 6 protein fractions have been tested *in vitro* and showed inhibitory effects on PHA or ConA stimulated bovine peripheral blood mononuclear cells. Two of these fractions exerted strong inhibitory effects (Lazarević et al. 1994.). It has been well documented in the mammalian species tested, that different SP molecules had inhibitory effects on the *in vitro* proliferation of their mononuclear cells. In humans, a dominant role is attributed to seminal PGE_2_ (Kelly 1988; Skibinski et al. 1992a), while in boars and rams inhibition is induced by an enzyme-seminal ribonuclease. In addition, this enzyme, originating from the bovine seminal vesicles is responsible for embryo toxic and cancerostatic activity of bovine SP as reviewed by Matoušek (1985). Also, Vukotić and Pavlović (1981) showed that component(s) of bovine SP can bind to the chromatin of a wide variety of cell types. The extracellular organelles prostasomes isolated from human SP may modulate activity of phagocytic cells (Skibinski et al. 1992b). We have demonstrated that both prostasomes and similar organelles vesiculosomes, isolated from bovine SP inhibited mitogen induced proliferation of human and bovine PBL in a dose dependent fashion and also phagocytosis of latex particles by bovine neutrophils. Additionally, superoxide production induced by suboptimal doses of phorbol ester and chemotactic peptide was decreased (Lazarević et al. 1995). A bulk data collected about prostasomes, indicated that they are among the important extracellular organelles in body fluids of man and animals as recently reviewed in detail by Vickram et al. (2020). One of probably the most important roles of human SP is its potential to inhibit the complement activation (Peterson et al. 1980). This finding was confirmed for bovine SP as well (Lazarević and Miletić 1990). Such phenomenon might be of a high biological importance. Namely, if the ASA are even produced, they will not activate the complement system with possible deleterious effects and the cell membrane attack complex will not be formed. On the other hand, there is evidence that bovine follicular fluid contains all components of the complement system and that its activity is significantly higher during oestrus. Total haemolytic follicular fluid complement activity is 2-22 times higher than that in serum (Fahmi and Hunter 1985). In the CM of woman, the haemolytic complement activity was also observed and considered regarding infertility in women with complement-dependent sperm immobilizing antibodies (Price and Boettcher 1979). Sperm cells are capable of activating complement by alternative pathway, and thus initialling the acrosomal reaction (Fahmi and Hunter 1985). It should be also taken into account in the AI of cows, the potential fact that the semen extenders may influence such activation. Accordingly, in order to find out if the mixture of bovine SP and egg yolk based semen extender (EYE) have the same effect on the *in vitro* stimulation of bovine lymphocytes by polyclonal mitogens (PHA and ConA), detailed investigations have been performed. The proliferation of PBL induced by PHA was inhibited by both bovine SP and SP-EYE. Interestingly, the EYE alone did not reduce the *in vitro* proliferation of bovine PBL. Conversely, the response in ConA stimulated PBL cultures was strongly inhibited by EYE and bovine SP-EYE mixture and to some extent by bovine SP. No cytotoxicity effect was recorded. When comparing the effects of the same volumes of bovine SP and its mixture with EYE, differences were highly significant due to the dilution grade. In the AI amount of the mixture of bovine SP and EYE is much smaller because the volume of the insemination straws is only 0.25–0.45** **mL as compared to the native ejaculate volume of 5–7** **mL which is deposited in cow’s vagina in the natural mating. By multiplying the grade of ejaculate dilution and the numerical outcomes calculated by dividing ejaculate and straw volumes, it could be concluded that degree of physiological immunosuppression needed to avoid the immune response of female to sperm antigens may not be ignored (Lazarević et al. 1992).

Furthermore, it has been reported that bovine SP inhibits precipitation of the immune complexes in a dose dependent fashion (Lazarević and Miletić 1993). This property was earlier attributed to complement and anti-Ig antibodies (Schifferly et al. 1982). The biological significance of this SP role is still unclear. In the procedure of preparing ejaculate for deep freezing, bovine SP is diluted several times and numerous new antigens have been added as constituents of the semen extenders. The use of the EYEs provided an excellent cryoprotection of sperm cells, but at the same time such extenders commonly contain numerous antigens encountered by the immune system of the male, and as well the specific antibodies by which the male responded to these antigens. The presence of the ASA in the sera of cows and heifers has been monitored and it was concluded that the titers of the ASA are increasing with the number of the AI. In order to study this phenomenon the sperm-agglutination test, despite the fact of being a very old method, proved to be highly reproducible and moreover one of the deleterious effects of the ASA is the agglutination. It has been also observed that spermatozoa differs in antigenic structure depending on the semen extender used (Jaćević et al. 1999).

However, a more precise indirect immunofluorescence method has been introduced latter on (Lazarević et al. 2003; Milovanović et al. 2005), which enabled the examination of both sera and CM in heifers and cows focusing on so called repeat breeder (RB) animals.

In the reproduction of dairy cows RB is still a significant problem. It is usually defined as a failure of the conception following three subsequent AI attempts in the animals without any symptoms of genital tract diseases. The general immune responsiveness was estimated by the cutaneous basophil hypersensitivity reaction (CBHR) to PHA mitogen in cows with different reproductive results, including the RB. Altogether, data regarding the hypersensitivity test presented herein were in agreement with the previous findings obtained by the CBHR testing on a smaller groups of cows and heifers, namely that the age of the cows seemed to have higher impact on the observed phenomenon rather than their reproductive history (Lazarević et al. 2006).

It was recorded that in sera and CM, higher levels of IgA class of the ASA were found in the cows with longer open days period and that their titres increase with the number of AI attempts, and therefore as well with the age. The repeated breeding is one of the major problems in cattle breeding, especially prominent in high yielding dairy cows. Namely, it is evident that these animals are healthy, with regular cycling and without pathology of genital tract, but somehow failed to be fertilized by the AI. In general, a RB is a cow which shows a reduced probability of conception while all other factors are optimal (Casida 1961). In order to find out if the RB cows are animals of generally low immune responsiveness it has been performed a cutaneous basophil hypersensitivity reaction, by testing a rather large population of the RB cows, but no consistent results were obtained (Lazarević et al. 2004a). The importance of the ASA for spermatozoa penetration through bovine CM was reported by Tas et al. (2007). Vikrant et al. (2016) conducted an interesting study in order to determine the levels of the ASA in blood serum and CM of the AI crossbred cows in the relation to their infertility. Although these authors have used entirely different assays (immunoperoxidase assay, SpermMar test and ELISA), their results are generally in agreement with our findings (Lazarević et al. 2003; Milovanović et al. 2005). In natural mating reproductive results of the RB have been significantly improved, and the conception rate was doubled in both heifers and cows (Vukotić and Pavlović 1986). Thus, these animals should be considered rather as sub-fertile then infertile. The authors also suggested that the RB is, at least partly, a consequence of deficiency of the bovine SP inhibitory activity. It could be hypothesized that if such cows are being AI following calving in the period when they are in a negative energy balance because of the lactation peak and when their chances to achieve pregnancy are by any means reduced due to the hormonal homeorhesis misbalance. It seems that farmers are somehow "forcing" animals to conceive at not proper time, increasing a number of the AI attempts needed for the conception, and thus inducing, rather weak, but important immune response to sperm antigens. On the contrary to these findings, scientists were not always able to establish a clear correlation between the ASA titres and poor reproductive results of cow. According to their opinion, the most studies conducted, three major mistakes have been usually made. Firstly, the blood sera of cows were examined and the ASA titres were increasing with age and number of the AI, but not always. Secondly, the investigators have been used spermatozoa from the native ejaculates, not from the AI straws, although they differ in genetic profiles and antigenic composition. In fact, the immune system of cow "never met" native spermatozoa and finally, only IgG class of the ASA titres or concentrations were measured. More recently, it has been undoubtedly demonstrated by the computer assisted sperm analyses method, that the samples of the CM containing high levels of the ASA of IgG, and especially of IgA class significantly reduced the parameters of spermatozoa motility, such as the total and the progressive motility. This was not a case if the effects of IgG and IgA classes of the ASA originated from the sera of cows were examined (Lazarević et al. 2013). Taken altogether, it might be concluded that when discussing possible negative immunological consequences of the AI, at least in cows, the ASA in the CM must be always considered. It is important to point out that such findings should be evaluated very carefully because the CM samples were collected during the oestrus when they may contain different amounts of water and, therefore were diluted to different extent influencing the final results.

The hypothesis of protective role of the SP for sperm cells from immunological destruction by female has been supported by many in vitro studies, and has put forward a novel aspect of the secretory activity of accessory sex glands, emphasizing their immunomodulatory activity as a primary role in the process of reproduction of viviparous mammals. Data for *in vivo* evidence supporting this hypothesis are few. Thus, the AI of cows and heifers could be a suitable animal model for evaluation of this hypothesis by observing the variations in the conception rate dependent on the number of inseminations. Also, the reactivity of sera of heifers to sperm cells and the comparison of the efficiency of the AI and natural mating in the RB animals. It has been found that the probability of the conception declined with the repeated AI as the reactivity of female sera to sperm cells increased. The superiority of the natural breeding over the AI could be explained by a protective role of the SP as proposed by the hypothesis (Vukotić and Pavlović 1986).

It is well known that bovine seminal plasma (BSP) contains potent immunosuppressive substances to prevent or minimize hypersensitization of females with the alloantigen’s on the surface of spermatozoa and in the seminal plasma itself. The effects of the BSP, egg yolk extender (EYE) and their mixture (BSP-EYE) on bovine PBL responses to the *in vitro* stimulation by either PHA or ConA mitogens have been studied. The PHA-induced proliferation of bovine PBL was decreased by the BSP and by the BSP-EYE mixture in a dose dependent mode while the EYE had no effect. The ConA-induced PBL response was strongly decreased by the EYE and the BSP-EYE mixture depending on a dose, and to some extent by the BSP. No cytotoxic effects were observed in the cultures of bovine PBL. By comparison of the effects of the same concentrations of the BSP and the BSP-EYE mixture a higher intensity of suppression of the BSP have been recorded (Lazarević et al. 1992).

The influence of the BSP on the immune (antigen-antibody) complexes precipitation has been investigated using kinetic nephelometric procedure. Also, the active substance(s) characterization was partly performed by the chromatography and the gel-filtration separation procedures. The formation of the ovalbumin (OVA/anti-OVA immune complexes has been inhibited by the BSP in a dose dependent manner showing almost linear correlation (r** **=** **0.997). The inhibitory activity of the BSP towards the immune complexes lattice formation, *i.e.,* precipitation it is an important biological phenomenon caused by mammalian SP. However, it would be of substantial interest to determine if the SP either inhibits the primary antigen-antibody reactions or the secondary large lattice formation-reaction only (Lazarević and Miletić 1993).

In order to separate protein molecules from the BSP and to investigate their influence on bovine PBL blastogenesis, the ion exchange DEAE Sephacel column chromatography has been performed. Pooled BSP was fractionated by the chromatography and 6 fractions were obtained. Their potential influence on bovine PBL blastogenesis was studied *in vitro* in cell cultures of the PBL stimulated with suboptimal doses of either PHA or ConA mitogens. The protein molecules that were present in the fractions 1 and 6 acted inhibitory in the PHA stimulated cultures. The fraction 1 was also inhibitory for the cultures of bovine PBL stimulated with the ConA. Further, the cell cultures stimulated with the PHA showed to be additionally stimulated with the protein molecules from the fractions 2 and 4. When the blastogenesis of bovine PBL was induced by the ConA only molecules from the fraction 4 stimulated cell proliferation. In all tests performed pooled SP exerted very strong inhibitory effects on mitogen induced bovine PBL blastogenesis (Lazarević et al. 1994).

The extracellular secretory vesicles isolated from bovine seminal plasma have immunomodulatory properties as it has been demonstrated. These vesicles inhibited the mitogen (PHA) induced proliferation of bovine and human PBL in a dose dependent fashion. Also, the vesicles inhibited the phagocytosis of latex particles by bovine neutrophils. The phagocytosis of opsonized *Staphylococcus aureus*, however, was not affected. Furthermore, phorbol ester and chemotactic peptide induced superoxide production was decreased especially when a suboptimal dose of the stimulants was used. It seems very likely that extracellular secretory vesicles may preserve bovine sperm survival in the female reproductive tract (Lazarević et al. 1995).

The evaluation of the methods for isolation of extracellular vesicles – vesiculosomes originating from seminal plasma vesicles of bulls was performed. Also, their effect on the proliferative *in vitro* responses of bovine and human PBL to PHA mitogen has been investigated. Comparatively, the effect of prostasomes – extracellular vesicles originating from the human prostate was tested in the same model system. It has been established that both vesiculosomes and prostasomes exhibited strong inhibitory effects on the proliferative responses of both human and bovine PBL pre-treated with the PHA. The observed effects were dose-dependent, and exhibited even after the extracellular vesicles have been exposed to high temperatures, indicating that such effects were not enzyme-mediated (Lazarević 1996).

A chronological presentation of the results obtained in the studies of the presence of sperm agglutinins in the blood serum and the CM of domestic, and some laboratory animals have been analyzed. A special attention was given to the consideration of the importance of the ASA and antibodies to antigens of sperm dilutants in the aetiopathogenesis of non-symptomatic infertility in heifers and cows. The evidences in a favour of the assumption that antibodies which may interfere with the successful reproduction can be present during the AI of heifers and cows (Lazarević and Jaćević 1998).

The existence of the antigenic differences between the native bull spermatozoa and those suspended in different extenders has been demonstrated. Furthermore, the validity of the semi quantitative KBM (Kibrick et al. 1952) gelatine agglutination test has been confirmed for detecting such antigenic changes. The elevation of the sperm agglutinin titre of antibodies in the blood sera and CM of the inseminated (bull semen diluted either in TRIS-EYE or in the new Biociphos plus extender, without animal proteins) (IMV, L'Aigle, France) heifers and cows correlated with the number of the AI, as have been stated by others also. However, the sperm agglutinin titre in the CM was significantly lower in heifers inseminated once than in the cows inseminated twice (*P*** **<** **0.01). As in the blood serum the sperm agglutinin titre was constantly higher in cows than in heifers, regardless the current finding which may contribute to the explanation of the roles of local and systemic immunity in the development of asymptomatic sterility in, at least domestic bovines (Jaćević et al. 1999).

The presence of antisperm antibodies in the sera of neonatal and young calves up to the age of 120** **days was detected by the IIF and the sperm-agglutination methods. By the IIF assay, anti IgG and IgM antibodies were detected. In the sera of neonatal calves, before colostrum ingestion, no anti-sperm antibodies were detected due to the physiological agammaglobulinemia. Titre obtained values in two day old and older calves were different for native sperm cells and sperm cells previously suspended in TRIS-EYE or “Biociphos plus” extender indicating antigenic differences between them. Their titre increased with age. Also, the hypothesis has been confirmed that in calves antisperm antibodies, naturally occurring before puberty, are the most probable consequence of cross reactivity with microbial antigens (Lazarević et al. 2002).

The levels of the IgA and IgG class ASA in the CM and sera of the AI cows have been determined by the IIF (indirect immunofluorescence) on the day of the AI. Sperm cells were suspended in the TRIS-EYE or «Biociphos +» extender. The mean titre values of the IgA ASA were significantly higher in the CM samples than in the sera and elevated with the number of the AI, indicating that the local mucosal immune response was of more relevance for the assessment of the immunological reactivity to sperm and extender antigens. The titer of the IgA class ASA was higher when the sperm cells suspended in TRIS- EYE were used for the test (Lazarević et al. 2003, Lazarević et al. 2004a). The titers of the IgG antibodies in CM were mostly below the sensitivity of the method (Lazarević et al. 2004b).

The titers of the ASA of IgG and IgA class in sera and CM of the AI Holstein cows were determined in order to correlate these results with the duration of the open days period. Blood and CM samples were collected on the day of the last AI and presence of the ASA was determined by IIF method using bulls sperm cells suspended in two above mentioned extenders. Such findings strongly confirmed the hypothesis that immune mechanisms may be involved in reproductive disturbances due to high levels of the ASA of IgA class. In the sera and CM of cows, high levels of the ASA were found in animals with longer open days period (Milovanović et al. 2005). Titres of total ASA determined by IIF increased with the number of AI. This was predominantly due to the increased titer of the IgA class ASA, while the titres of the IgG class did not show any significant differences. The ASA of the IgG class have no important role in the humoral immune responses induced at the mucosal surfaces of the genital tract (Milovanović et al. 2008).

**Figure 1.**
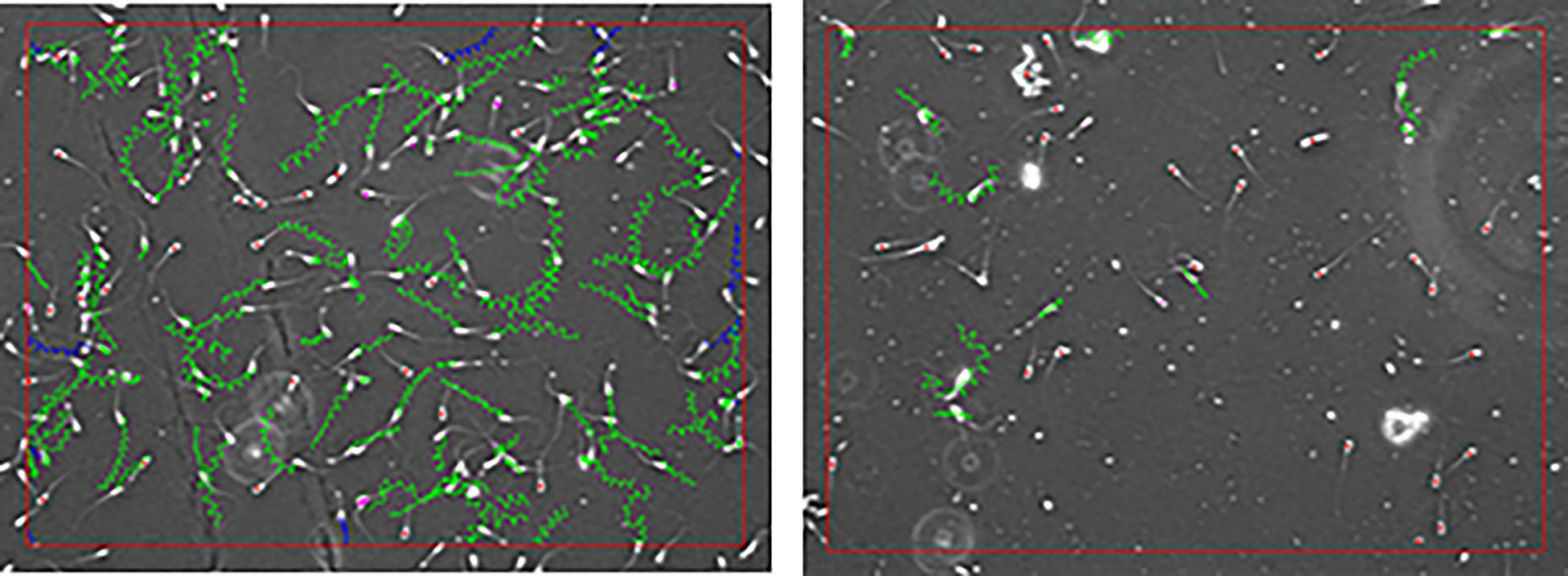
Pictures acquired by CASA on the Hamilton - Thorn (HTM IVOS Version 2, No M 9368, USA) sperm analyser (magnification 100 x): a. Control sample with bull's sperm cells suspension and low Ig A class ASA titer in cervical mucus; b. Sample with the same bull's sperm cells suspension and high Ig A class ASA titer in cervical mucus. Curved green lines represent sperm cells expressing progressive motility.

This research was continued by investigating the influence of the ASA of IgG and IgA class from the sera and CM of the Busha breed or mixed breed with the Simmental breed cows on motility of bull’s sperm. The presence of the IgG and IgA ASA was determined by the IIF and cows were divided into groups with high or low Ig G and Ig A ASA levels. Sperm motility was estimated by the Computer Assisted Semen Analysis (CASA). The significant differences in the influence of the ASA depending on their origin and titre on the sperm motility were established. The levels of the ASA increased with the age and a total number of the AI attempts. The ASA of the Ig A class in the CM significantly reduced sperm motility (Figure 1a, b), and thus possibly may decrease chances for successful fertilization (Lazarević et al. 2013).

There is little evidence for a decline in fertility when a well-designed AI scheme is implemented. Pregnancy rates to the AI similar to natural mating have been observed for beef and dairy cattle, pigs and horses, therefore, almost for all livestock animals of veterinary importance (Hansen 2011). In the animals which are important for livestock production, as well as in recreation animals, particular knowledge may be applied to health and breeding programs. Recently, in the field of the ART remarkable progress has been made, particularly in: AI, embryo transfer, *in vitro* fertilization, embryo cryopreservation, sexing of semen and embryos, cloning, transgenesis, stem cell technology, embryo genomics, and micro- and nanotechnology. Nonetheless, imperfections are remaining and the sustained efforts will be required to optimize existing and invent new technologies. Before referring an animal for the use of an ART, veterinary practitioners should be able to identify the underlying cause of subfertility of that animal. Knowing the complexity as well as the risks of these techniques, this enables practitioners to refer a subfertile animal to the least complex and most appropriate and successful ART that can overcome specific causes of infertility (Verma et al. 2012). The use of the ART has helped farmers to produce offspring from valuable purebred and autochthonous crossbred animals that were considered infertile using standard breeding techniques (Robertson et al. 2009). The resistance of domestic animals to disease might also be improved through the ART by genetic selection of animals for the immunological traits. Furthermore, such ART, based on application of immunological principles for the treatment of infertility and pregnancy loss has potential to improve pregnancy rates as well as the M-F health (Young 2016).

Instead of the conclusion or epilogue it seems worthwhile to evaluate briefly the compatibility of the contents of the former paragraph, which is focused on the reproductive immunology of bovines in relation to humans (only for the *in vitro* testing), with the basic principles of the veterinary immunology, the area of general immunology, also including human immunology. Namely, veterinary immunology, defined as the immunology of domestic and wild animals having economical or sentimental value to man, provides both practical knowledge that is useful to animal husbandry (animal breeding/nutrition), and new insights into fundamental immunology (animal immunogenetics/health programs). The fulfilment of these objectives benefits from the flow of information and communications among immunologists working on domestic animals. In addition to national specialized groups, the Veterinary Immunology Committee of the International Union of Immunological Societies (VIC-IUIS) was established to facilitate communication on veterinary immunology, and to establish 'official' links between veterinary immunology and the rest of the immunologists' community. Finally, it should be mentioned the motto of veterinary immunologists accepted in the mid-1980s by the VIC of the IUIS as follows: “Veterinary immunology considers an animal the aim not the tool of research” (Charley et al. 1996).

## 7. Immunological manipulation of the mammalian reproductive system

The immune system in higher organisms, like mammals may act as friend or foe. As such, pregnancy has been long considered as a state of relative immunosuppression or tolerance of semiallogeneic foetus, which is not synonymous for generalized immunologic unresponsiveness. Actually, it is antigen-specific and causes no impairment of the immune response to antigens other than the ones that induce tolerance. Now, it is recognized that normal pregnancy is not a state of total immunosuppression but rather a state of dramatic systemic and local hormonal and metabolic alterations mediating immune modulation of the maternal immune system and immune responses, including those to foetal paternal antigens (Kutteh et al. 2019). Attempts to increase reproductive performance in domestic farm animals include stimulation of reproductive hormone secretion, induction of ovulation and superovulation, increasing of fecundity, and control of fertility (Bhardwaj et al. 2012) using purified and synthetic hormones, and more recently, the use of *in vitro* fertilization and embryo transfer techniques. These methodologies have provided various degrees of success in increasing reproductive performance in different mammalian species. The most commonly applied active immunization by raising antibodies against reproductive hormones has been used to explore potential for augmenting reproductive efficiency in livestock, and was considered as to be more practical than a passive immunization. Reproductive consequences have been studied following immunization against: (i) reproductive hormones (luteinizing hormone releasing hormone, gonadotrophic hormones, gonadal steroids, oxytocin, PGF_2α_, and inhibin), (ii) gamete antigens (sperm antigens and *zona pellucida* antigens), and (iii) conceptus, in laboratory animals (mouse, rat, and rabbit), domestic food mammals (pigs, cattle, sheep, and goats) and domestic avian species (poultry), domestic companion animals (dogs and horses), wild animals (elks, elephants, and monkeys) as well as in humans (Yitbarek and Regasa 2014).

### 7.1. Immunological therapy and biotechnological methods for treating infertility or recurrent miscarriages

Many studies have suggested that women with pathological pregnancies such as the RSA have signs of generally excessive inflammatory immune responses both before and during pregnancy, and signs of breakage of tolerance to autoantigens and foetal antigens. In addition, several abnormalities of the innate immune responses involve disadvantageous interactions between uNK cells and HLA-G and HLA-C on the trophoblast that may have proinflammatory effects and increased level of inflammation at the M-F interface predisposing to adaptive immune processes against alloantigen’s on the trophoblast that may pose the risk of pathological pregnancy outcome (Christiansen 2013). Accordingly, if the major mechanism in the RSA of unknown ethology is immunological, manipulation of the immune system may be an appropriate and efficacious therapeutic approach (Chen et al. 2012). Few approaches to manipulate the immune response of the mother to the foetus have been employed. First attempts included immunization of the woman either with husbands or with third-party lymphocytes, but very few systematic clinical studies testing the efficacy of such treatment have been reported. Predominantly, these studies have shown no significant beneficial effect over placebo in improving the live birth rate in the RSA couples (Jeve and Davies 2014). Several therapeutic approaches have been used to prevent RSA by modulating immune response during pregnancy (Chen et al. 2012). Mostly, human Ig has been widely used in the treatment of RSA especially for unexplained and poor outcome cases, (Jeve and Davies 2014). Human chorionic gonadotropin hormone (hCG) has been also used as a basic agent for the treatment of miscarriage (Schumacher 2017). Although its therapeutic mechanism is not fully understood, the hCG plays an active role in the prevention and treatment of the RSA (Makrigiannakis et al. 2011). Recent study investigated the treatment of RSA patients with human Ig combined with hCG (Guo et al. 2020). Changes in the expression levels of Th17 and Treg cells before and after treatment were compared with values determined in control patients with successful pregnancy. In the RSA patients, the proportion of Th17 cells in peripheral blood and decidual tissue was increased and the proportion of Treg cells decreased. After the treatment, the proportion of Th17 cells in patients decreased and Treg cells increased, *i.e.,* the Th17 and Treg cells balance was reversed. This study suggested that the Th17 and Treg cell immune imbalance may be an important immune factor in the RSA, and combined Ig + hCG therapy may improve pregnancy outcomes in the RSA patients by reversing the imbalance between Th17 and Treg cells. Thus, if unexplained RSA are associated with such immune dysfunction at the M-F interface, an immune endocrine therapy with combination of Ig and HCG may revise this immune imbalance in women experiencing the RSA and thereby increase the clinical pregnancy rates. However, the effectiveness of this treatment requires further investigation. Namely, immunological therapy is a relatively novel approach for treating cases of unexplained infertility or recurrent miscarriages mostly in humans. The use of different lines of immune therapies (such as prednisolone, intravenous Ig, intralipid, TNF-α blockers), to decrease the activity of uNK cells in infertile/subfertile patients, has to be reconsidered because the scientific principle of the M-F tolerance has been misunderstood (Lewis et al. 1986). In recent decades, substantial progress has been achieved to improve the assisted reproductive treatments of recurrent miscarriages and recurrent implantation failure due to new insights of the role of the immune system in maternal immune tolerance to pregnancy (Young 2016). Following the discovery of the reproductive hormone system, and understanding of their roles, the attempts were made to control reproduction by active immunization against the key hormones in animals. Recently, such vaccines have found applications in animal reproduction processes (Yitbarek and Regasa 2014). Although seven decades passed after the first live calf was born following embryo transfer performed by Elwyn Willet and colleagues in Madison, WI, USA, in 1950 (Hansen 2010), still the extensive research has been conducted in domestic animals, particularly in cattle. The reproductive procedures of sperm handling, capacitation, and acrosome reaction, superovulation, and embryo handling, sexing, bisection, cryopreservation, and transfer are now in practical use. Because of the economic importance of cattle such biotechnology has been tested and improved under clinical conditions. The large number of normal progeny produced in cattle after a long prenatal development period, similar to humans, provides some assurance that this biotechnology, carefully applied, is safe for both domestic animals and humans (Foote 1987).

### 7.2. Improvement of reproductive performances in domestic animals by exogenous immunomodulators

Improvement of reproductive performance and fertility/fecundity management are of great importance in livestock production. Immunomodulation is generally described as pharmacological manipulation of the immune system by application of a variety of natural and/or synthetic immunomodulatory substances (Gokuldas et al. 2010). Regulation of reproduction in domestic animals by immune intervention, such as immunomodulation is a recent and tolerably approach (coinciding with the ban of dietary antibiotic growth promoters in production of food animals), only if applied immunomodulatory substances are proven to be harmless for health of animals and humans as well as unhazardous for the environment.

The reproductive performance of gilts transported for long distances from breeding farms to commercial farms was tested after the application of an immunomodulator (IM) Baypamun N® (Bayer, Leverkusen, Germany), based on inactivated *Parapoxvirus ovis* strain D1701. The treatment significantly increased the proportion of gilts farrowed/total gilts tested, the total number of piglets born, the number of those born alive and their body weight at farrowing (Saratsis et al. 1999). Similar findings were subsequently reported for the pregnant gilts, grown under commercial farm conditions and moved from the sow facility unit to the farrowing unit following the treatment with a novel formulation of nonspecific IM, Baypamun**©** (Bayer, Leverkusen, Germany). As such, the reproductive performance was enhanced since the numbers of liveborn and stillborn piglets farrowed to the treated gilts were significantly either higher or lower, respectively, than those recorded in the nontreated controls (Potočnjak et al. 2006). For a rather long time well known as an effective IM, a synthetic antihelmintic medication levamisole has been studied for its influence on the improvement of reproductive efficiency in boars without inducing adverse effects on immunological, haematological and serum biochemistry parameters. The 3-day treatment with levamisole resulted in significantly elevated sperm parameters, such as volume and total number of sperm doses, total number of sperm cells, their density and motility as determined for 49** **days at weekly basis. Also, tested parameters of systemic humoral and cellular immunity such as total immunoglobulins and neopterins as well as number of leukocytes and monocytes were increased, with only exception of slightly decreased thrombocytes (Samardžija et al. 2008). Exogenous melatonin induced a significant reduction of the number and rate of non-viable embryos and improved the survival of embryos collected from ewes after superovulation in anoestrus. It appears that the uterine sensitivity to progesterone – in terms of progesterone receptor expression - could be reduced by melatonin treatment of sheep. It seems that melatonin increased lamb production by more expressed luteotrophic effect and improved embryonic survival (Abecia et al. 2008). Interestingly, homeopathic preparation Traumeel^®^ (Biologische Heilmittel Heel GmbH, Baden Baden, Germany), naturally derived agent (comprising extracts from 12 aromatic plant species, one mineral, and one animal-originated substance, *i.e.,* Ca-oxide from the oyster shell) has been evaluated as a potential IM for enhancing immune and reproductive system parameters in breeding boars. The 4-days treatment of boars with the preparation induced significantly higher average sperm volume and the sperm cells motility, but did not affect the immune parameters tested, except slightly but significantly higher level of γ-globulin fraction of total serum immunoglobulins as recorded weekly for 7** **weeks before and after the treatment (Marković et al. 2008). The combined therapy of *Escherichia coli* lipopolysaccharide, an IM, along with administration of oxytocin after mating, applied as an alternative therapy for mares with persistent endometritis has been effective for the elimination of uterine inflammation and for the improvement of reproductive performance through an increase of pregnancy and foaling rates in subfertile mares (Sharma and Dhaliwal 2010). The 2-days treatment of breeding bulls with the preparation of inactivated *Parapoxvirus ovis* strain D1701 (former trade name Baypamun^®^, and now Zylexis^®^, Pfizer Animal Health, Louvain-la- Neuve, Belgium), has been performed to provide data on its effect on immunity and sperm quality in Simmental bulls exposed to permanent stress conditions. The positive effects of the IM applied have been observed as serum cortisol levels were decreased at day 8 and day 12 after treatment of the bulls. The parameters of the reproductive efficiency such as sperm concentration in ejaculate, number of sperm doses per ejaculate, and motility of frozen/thawed sperm cells were significantly increased. It is very likely that the IM tested diminished negative influence of stress by decreasing the level of cortisol in the peripheral blood of breeding bulls, and simultaneously positively influenced abovementioned parameters of reproductive efficiency. None of immunohematological parameters tested were changed by the IM applied (Marković et al. 2010). More recently, novel preparations with putative properties of an IM have been introduced in prevention and control of pregnancy disorders. Metallothioneins (MT), the cysteine-rich proteins are implicated in regulation of physiological processes, such as cell growth and differentiation, repair and apoptosis, and immunoregulation, as well as in the protection against heavy metals, oxidant damages, inflammation and other stressful conditions. Their favourable potentials were tested at the maternofoetal interface and in foetal organogenesis by monitoring the tissue expression of MT I/II isoforms in murine undisturbed syngeneic pregnancy, after the treatment with an IM of bacterial origin, peptidoglycan monomer linked with zinc (PGM-Zn). The treatment with the PGM-Zn preparation markedly enhanced the intensity of MT tissue expression in placenta and trophoblast cells, and particularly in the foetal organs. The data imply that MT are involved in the protection of trophoblast cells against the pregnancy-induced deregulation of redox and neuro-immuno-endocrine homeostasis, in transport and storage of essential metals required for foetal organogenesis, as well as in the protection of foetus against bacterial toxins (Jakovac et al. 2015). Knowledge about a vast variety of natural (endogenous and exogenous) or synthetic substances with immunomodulatory properties which may act as IM to maintain an optimally effective, but not excessive and harmless innate and adaptive immune responses to foetal and trophoblast antigens is crucial in order to develop an efficient immune therapy to patients with previous early and late pregnancy complications. If applicable in practice, this will bring into reproductive immunology the same type of curative revolution as antibiotics have in the combat against infectious diseases of bacterial aetiology.

Considerable efforts have been focused to understanding of mammalian reproductive system infectious diseases, their diagnosis, including biology of pathogens, host resistance and therapy in the production of farm animals. Conversely, little is known on prevention of such diseases through immunomodulatory and dietary strategies because these problems have been overcome by adding the sub-therapeutic doses of in-feed antibiotic growth promoters (AGP). However, since 2006 the European-wide directives are restricting the non-clinical use of AGP in food animal production, and thus, we should consider the efficiency of IM.

## Abbreviations

(AIDS): acquired immunodeficiency syndrome
(APC): antigen presenting cells
(ANA): antinuclear antibodies
(APL): antiphospholipid
(ASA): antisperm antibodies
(ATA): antithyroid antibodies
(ART): assisted reproductive technologies
(CM): cervical mucus
(CSF2): colony stimulating factor 2
(CASA): Computer Assisted Semen Analysis
(CRH): corticotrophin-releasing hormone
(CBHR): cutaneous basophil hypersensitivity reaction
(DM): decidual macrophages
(DC): dendritic cells
(EYE): egg yolk extender
(FasL): Fas/Fas ligand
(GnRH): gonadotropin releasing hormone
(GNLY): granulysin
(HY): histocompatibility
(H-antigens): histocompatibility allantigens
(hCG): human chorionic gonadotropin hormone
(HLA): human leukocyte antigens
(IM): immunomodulator
(LH): luteinizing hormone
(LAK): lymphokine-activated killer
(MHC): major histocompatibility complex
(MHS): major histocompatibility system
(MBL): mannose-binding lectin
(M-F): maternofoetal
(MT): metallothioneins
(mya): million years ago
(PGM-Zn): peptidoglycan monomer linked with zinc
(PBL): peripheral blood leukocytes
(PE): preeclampsia
(PGE2): prostaglandin E2
(PG): prostaglandins
(PTPRC): protein tyrosine phosphatase, receptor type C
(Rag): recombination-activating genes
(RPL): recurrent pregnancy loss
(RSA): recurrent spontaneous abortion
(SP): seminal plasma
(TCR): T cell receptor
(TNFα): tumour necrosis factor alpha;
(TAG-72): tumour-associated glycoprotein-72
(uNK): uterine natural killer
(VIC-IUIS): Veterinary Immunology Committee of the International Union of Immunological Societies

## References

[CIT0001] Abecia JA, Forcada F, Casao A, Palacin I. 2008 Effect of exogenous melatonin on the ovary, the embryo and the establishment of pregnancy in sheep. Animal. 2(3):399–404. 22445042 10.1017/S1751731107001383

[CIT0002] Amos DB. 1968 Human histocompatibility locus HL-A. Science. 159(3815):659–660. 10.1126/science.159.3815.659-a 4886902

[CIT0003] Anderson D, Billingham RE, Lampkin GH, Medawar PB. 1951 The use of skin grafting to distinguish between monozygotic and dizygotic twins in cattle. Heredity. 5(3):379–397.

[CIT0004] Antczak DF. 1982 Structure and function of the major histocompatibility complex in domestic animals. J Am Vet Med Assoc. 181(10):1030–1036. 6816772

[CIT0005] Balner H, Gabb BW, Dersjant H, Van Vreeswijk W, Van Rood JJ. 1971 Major histocompatibility locus of rhesus monkeys (RhL-A)). Nat New Biol. 230(14):177–180. 4995128 10.1038/newbio230177a0

[CIT0006] Balner H, Van Vreeswijk W, Roger JH, D'amaro J. 1978 The major histocompatibility complex of chimpanzees: Identification of several new antigens controlled by the A and B loci of ChLA. Tissue Antigens. 12(1):1–18. 694913

[CIT0007] Barker CF, Markmann JF. 2013 Historical overview of transplantation. Cold Spring Harb Perspect Med. 3(4):a014977 23545575 10.1101/cshperspect.a014977 PMC3684003

[CIT0008] Beer AE, Quebbeman JF, Ayers JWT, Haines RF. 1981 Major histocompatibility complex antigens, maternal and paternal immune responses, and chronic habitual abortions in humans. Am J Obstet Gynecol. 141(8):987–997. 6459031 10.1016/s0002-9378(16)32690-4

[CIT0009] Bhardwaj A, Nayan V, P, Gupta M AK. 2012 Inhibin: a role for fecundity augmentation in farm animals. Asian J of Animal and Veterinary Advances. 7(9):771–789.

[CIT0010] Billingham RE, Beer AE. 1984 Reproductive immunology: past, present, and future. Perspect Biol Med. 27(2):259–275. 6371705 10.1353/pbm.1984.0042

[CIT0011] Billingham RE, Brent L, Medawar PB. 1953 Actively acquired tolerance of foreign cells. Nature. 172(4379):603–606. 13099277 10.1038/172603a0

[CIT0012] Blackwell JM, Jamieson SE, Burgner D. 2009 HLA and infectious diseases. Clin Microbiol Rev. 22(2):370–385. 19366919 10.1128/CMR.00048-08 PMC2668228

[CIT0013] Blois SM, Kammerer U, Soto CA, Tometten MC, Shaikly V, Barrientos G, Jurd R, Rukavina D, Thomson AW, Klapp BF, et al. 2007 Dendritic cells: key to fetal tolerance. Biol Reprod. 77(4):590–598. 17596562 10.1095/biolreprod.107.060632

[CIT0014] Bogović-Crnčić T, Laškarin G, Juretić K, Štrbo N, Dupor J, Sršen S, Randić L, Le Bouteiller P, Tabiasco J, Rukavina D. 2005 Perforin and Fas/FasL cytolytic pathways at the maternal-fetal interface. Am J Reprod Immunol. 54(5):241–248. 16212646 10.1111/j.1600-0897.2005.00320.x

[CIT0015] Bogović-Crnčić T, Laškarin G, Juretić-Franković K, Sotošek-Tokmadzić V, Štrbo N, Bedenicki I, Le Bouteiller P, Tabiasco J, Rukavina D. 2007 Early pregnancy decidual lymphocytes beside perforin use Fas ligand (FasL) mediated cytotoxicity. J Reprod Immunol. 73(2):108–117. 16950518 10.1016/j.jri.2006.07.001

[CIT0016] Caldwell J. 1979 Polymorphism of the BoLA system. Tissue Antigens. 13(5):319–326. 91208 10.1111/j.1399-0039.1979.tb00803.x

[CIT0017] Casida EL. 1961 Present status of the repeat breeder cow problem. J Dairy Sci. 44(12):2323–2329.

[CIT0018] Cervera R, Balasch J. 2010 Autoimmunity and recurrent pregnancy losses. Clin Rev Allergy Immunol. 39(3):148–152. 19842070 10.1007/s12016-009-8179-1

[CIT0019] Charley B. 1996 The immunology of domestic animals: its present and future. Vet Immunol Immunopathol. 54(1–4):3–6. 8988844 10.1016/s0165-2427(96)05681-4

[CIT0276] Chavatte-Palmer P, Tarrade A. 2016 Placentation in different mammalian species. Ann Endocrinol (Paris)). 77(2):67–74. 27155775 10.1016/j.ando.2016.04.006

[CIT0020] Chen SJ, Liu YL, Sytwu HK. 2012 Immunological regulation in pregnancy: from mechanism to therapeutic strategy for immunomodulation. Clin Dev Immunol. 2012:258391–10. 258391. 22110530 10.1155/2012/258391 PMC3216345

[CIT0021] Cheng L, Xin Y, Liu X, Hu X, Xiang M, Wang D, Zhao S. 2016 The relationship between progesterone and Th-related cytokines in plasma during early pregnancy in cows. Front Agr Sci Eng. 3(2):147–152.

[CIT0022] Christiansen OB. 2013 Reproductive immunology. Mol Immunol. 55(1):8–15. 23062611 10.1016/j.molimm.2012.08.025

[CIT0023] Clarke GN. 2009 Etiology of sperm immunity in women. Fertil Steril. 91(2):639–643. 18281044 10.1016/j.fertnstert.2007.11.045

[CIT0024] Counce S, Smith P, Barth R, Snell GD. 1956 Strong and weak histocompatibility gene differences in mice and their role in the rejection of homografts of tumors and skin. Ann Surg. 144(2):198–204. 13355191 10.1097/00000658-195608000-00009 PMC1465326

[CIT0025] Croy BA. 2014a Reproductive immunology issue one: cellular and molecular biology. Cell Mol Immunol. 11(5):405–406. 25066420 10.1038/cmi.2014.64 PMC4197211

[CIT0026] Croy BA. 2014b Reproductive immunology issue 2: cellular and molecular biology. Cell Mol Immunol. 11(6):503–505. 25263487 10.1038/cmi.2014.98 PMC4220845

[CIT0027] Dausset J, Nenna A, Brecy H. 1954 Leukoagglutinins. V. Leukoagglutinins in chronic idiopathic or symptomatic pancytopenia and in paroxysmal nocturnal hemoglobinuria. Blood. 9(7):696–720. 13172288

[CIT0028] Dausset J, Rapaport FT, Cannon FD, Ferrebee JW. 1971 Histocompatibility studies in a closely bred colony of dogs. Ill: genetic definition of the DLA system of canine histocompatibility, with particular reference to the comparative immunogenicity of the major transpantable organs. J Exp Med. 134(5):1222–1237. 4939370 10.1084/jem.134.5.1222 PMC2139009

[CIT0029] Dey SK, Lim H, Das SK, Reese J, Paria BC, Daikoku T, Wang H. 2004 Molecular cues to implantation. Endocr Rev. 25(3):341–373. 15180948 10.1210/er.2003-0020

[CIT0030] Dominović M, Rukavina D. 2011 Immune cells and cytokines at the maternal-fetal interface. Maced Med Rev. 65:73–78.

[CIT0031] Duncan WR, Streilein JW, Ivanyi P. 1977 The major histocompatibility system of Syrian hamster In: Götze D., editor, The major histocompatibility complex in man and animals. Springer-Verlag: Berlin Heidelberg; p. 183–184. ISBN 978-3-642-95293-7

[CIT0032] El Demiry M, James K. 1988 Lymphocyte subsets and macrophages in the male genital tract in health and disease. A monoclonal antibody-based study Eur Urol. 14(3):226–235. 3289938 10.1159/000472944

[CIT0033] Fahmi HA, Hunter AG, Markham RJF, Seguin BE. 1985a Immunosuppressive activity of bovine seminal plasma on bovine lymphocytes in Vitro. J Dairy Sci. 68(9):2315–2321. 2933433 10.3168/jds.S0022-0302(85)81105-X

[CIT0034] Fahmi HA, Hunter AG, Markham RJF, Seguin BE. 1985b Identification of an immunosuppressive protein in bovine seminal plasma with activity against bovine lymphocytes. J Dairy Sci. 68(9):2322–2328. 2933434 10.3168/jds.S0022-0302(85)81106-1

[CIT0035] Fahmi HA, Hunter AG. 1985 Effect of estrual stage on complement activity in bovine follicular fluid. J Dairy Sci. 68(12):3318–3322. 4093525 10.3168/jds.S0022-0302(85)81241-8

[CIT0036] Fahmi HA, Hunter AG. 1986 Individual variation in immunosuppressive activity of bovine seminal plasma on Concanavalin A stimulated bovine T - lymphocytes in Vitro. J Dairy Sci. 69(2):527–530. 3486200 10.3168/jds.S0022-0302(86)80432-5

[CIT0037] Figueiredo AS, Schumacher A. 2016 The T helper type 17/regulatory T cell paradigm in pregnancy. Immunology. 148(1):13–21. 26855005 10.1111/imm.12595 PMC4819144

[CIT0038] Foote RH. 1987 In vitro fertilization and embryo transfer in domestic animals: Applications in animals and implications for humans. J in Vitro Fert Embryo Transf. 4(2):73–88. 10.1007/BF01555444 3298486

[CIT0039] Franken DR, Slabber CF. 1981 Reproductive immunology: 1. The effect of different protein concentrations of seminal plasma on in vitro lymphocyte cultures. Andrologia. 13(5):504–507. 7316239 10.1111/j.1439-0272.1981.tb00091.x

[CIT0040] Fugmann SD. 2010 The origins of the RAG genes-from transposition to V(D)J recombination. Semin Immunol. 22(1):10–16. 20004590 10.1016/j.smim.2009.11.004 PMC2823946

[CIT0041] Geczy AF, De Weck AL, Schwartz BD, Shevach EM. 1975 The major histocompatibility complex of the guinea pig. I. Serologic and genetic studies. J Immunol. 115(6):1704–1710. 52675

[CIT0042] Geraghty DE, Koller BH, Hansen JA, Orr HT. 1992 The HLA class I gene family includes at least six genes and twelve pseudogenes and gene fragments. J Immunol. 149(6):1934–1946. 1517563

[CIT0043] Geraghty DE. 1993 Structure of the HLA class I region and expression of its resident genes. Curr Opin ImmunoI. 5(1):3–7. 10.1016/0952-7915(93)90073-2 8452672

[CIT0044] Gerenčer M, Dražančić A, Kuvačić I, Tomašković Z, Kaštelan A. 1979 HLA antigen studies in women with recurrent gestational disorders. Fertil Steril. 31(4):401–404. 428584

[CIT0045] Gerenčer M, Kaštelan A. 1983 The role of HLA-D region in feto-maternal interactions. Transplant Proc. 15:893–895.

[CIT0046] Gerenčer M, Singer Z, Pfeifer S, Tomašković M, Humar I, Mezulić V, Kuvačić I, Žepić L, Kaštelan A. 1988 HLA and red blood group antigens in pregnancy disorders. Tissue Antigens. 32(3):130–138. 3217929 10.1111/j.1399-0039.1988.tb01648.x

[CIT0047] Gokuldas PP, Pramod Kumar R, Aravind S, Sreejith JR, Rathish RL. 2010 Fertility management through immunomodulation. J Indian Vet Assoc. 8(1):57–61.

[CIT0048] Gorer PA. 1937 The genetic and antigenic basis of tumor transplantation. J Pathol. 44(3):691–697.

[CIT0049] Götze D. 2012 The major histocompatibility system In: Götze D., editor. The major histocompatibility system in man and animals. Springer - Verlag, Berlin Heidelberg, Germany, p. 1–6.

[CIT0050] Günther E, Stark O. 1979 The major histocompatibility system of the rat. Transplant Proc. 11(3):1550–1553. 92081

[CIT0051] Guo Z, Xu Y, Zheng Q, Liu Y, Liu X. 2020 Analysis of chromosomes and the T helper 17 and regulatory T cell balance in patients with recurrent spontaneous abortion. Exp Ther Med. 19(4):3159–3166. 32256804 10.3892/etm.2020.8537 PMC7086275

[CIT0052] Hăla K. 1977 The major histocompatibility complex of the chicken In: Götze D., editor. The major histocompatibility system in man and animals. Springer-Verlag Berlin Heidelberg; p. 291–312. ISBN 978-3-642-95295-1.

[CIT0053] Hansen PJ, Soto P, Natzke RP. 2004 Mastitis and fertility in cattle – possible involvement of inflammation or immune activation in embryonic mortality. Am J Reprod Immunol. 51(4):294–301. 15212683 10.1111/j.1600-0897.2004.00160.x

[CIT0054] Hansen PJ. 2010 Medawar redux - an overview on the use of farm animal models to elucidate principles of reproductive immunology. Am J Reprod Immunol. 64(4):225–230. 20678170 10.1111/j.1600-0897.2010.00900.x

[CIT0055] Hansen PJ. 2011 The immunology of early pregnancy in farm animals. Reprod Dom Anim. 46(s3):18–30. 10.1111/j.1439-0531.2011.01850.x 21854458

[CIT0056] Heitzeneder S, Seidel M, Förster-Waldl E, Heitger A. 2012 Mannan-binding lectin deficiency – good news, bad news, doesn't matter? Clin Immunol. 143(1):22–38. 22377282 10.1016/j.clim.2011.11.002

[CIT0057] Ho HN, Gill TJ, Nsieh RP, Hsieh HJ, Lee TY. 1990 Sharing of human leukocyte antigens in primary and secondary recurrent spontaneous abortions. Am J Obstet Gynecol. 163(1 Pt 1):178–188. 2165353 10.1016/s0002-9378(11)90696-6

[CIT0058] Houlihan JM, Biro PA, Fergar-Payne A, Simpson KL, Holmes CH. 1992 Evidence for the expression of non HLA-A,-B,-C class I genes in the hnman fetal liver. J Immunol. 149(2):668–675. 1624808

[CIT0059] Hunt JS, Petroff MG, McIntire RH, Ober C. 2005 HLA-G and immune tolerance in pregnancy. Faseb J. 19(7):681–693. 15857883 10.1096/fj.04-2078rev

[CIT0060] Hunt JS, Vassmer D, Ferguson TA, Miller L. 1997 Fas ligand is positioned in mouse uterus and placenta to prevent trafficking of activated leukocytes between the mother and the conceptus. J Immunol. 158(9):4122–4128. 9126971

[CIT0061] Hyde KJ, Schust DJ. 2016 Immunologic challenges of human reproduction: an evolving story. Fertil Steril. 106(3):499–510. 27477190 10.1016/j.fertnstert.2016.07.1073

[CIT0062] Jaćević V, Lazarević M, Jakovljević G, Subotin L, Jovičin M. 1999 Antibodies against bulls sperm cells in the blood serum and cervical mucus of heifers and cows. Maced Vet Rev. 28(1-2):7–22.

[CIT0063] Jakovac H, Grebić D, Mrakovčić-Šutić I, Rukavina D, Radošević-Stašić B. 2015 Expression of metallothioneins in placental and fetal tissues in undisturbed and PGM-Zn treated syngeneic pregnancy. AJBIO. 3(2):1–7.

[CIT0064] James K, Hargreave TB. 1984 Immunosuppression by seminal plasma and its possible clinical significance. Immunol Today. 5(12):357–362. 25290980 10.1016/0167-5699(84)90079-3

[CIT0065] Jeve YB, Davies W. 2014 Evidence-based management of recurrent miscarriages. J Hum Reprod Sci. 7(3):159–169. 25395740 10.4103/0974-1208.142475 PMC4229790

[CIT0066] Johnson MH, Setchell BP. 1968 Protein and immunoglobulin content of rete testis fluid of rams. J Reprod Fertil. 17(2):403–406. 4177664 10.1530/jrf.0.0170403

[CIT0067] Johnson PM, Stern PL. 1986 Antigen expression at human maternal-fetal interfaces. Prog Immunol. 6:1056–1069.

[CIT0068] Jonjić S, Pavić I, Lučin P, Rukavina D, Koszinowski UH. 1990 Efficacious control of cytomegalovirus infection after long-term depletion of CD8+ T lymphocytes. J Virol. 64(11):5457–5464. 1976821 10.1128/jvi.64.11.5457-5464.1990 PMC248597

[CIT0069] Juretić K, Štrbo N, Bogović-Crnčić T, Laškarin G, Rukavina D. 2004 An insight into the dendritic cells at the maternal-fetal interface. Am J Reprod Immunol. 52(6):350–355. 15663599 10.1111/j.1600-0897.2004.00232.x

[CIT0070] Kelly RN. 1988 Prostaglandins in semen. In: Eicosanoids and reproduction, Ed. M. D. Mitchel, CRC Press, Florida USA, p. 55–72.

[CIT0071] Kirszenbaum M, Moreau P, Gluckman E, Dausset J, Carosella E. 1994 An alternatively spliced form of HLA-G mRNA in human trophoblasts and evidence for the presence of HLA-G transcript in adult lymphocytes. Proc Natl Acad Sci U S A. 91(10):4209–4213. 8183892 10.1073/pnas.91.10.4209 PMC43754

[CIT0072] Kirszenbaum M, Moreau P, Teyssier M, Lafon C, Gluckman E, Dausset J, Carosella E. 1995 Evidence for the presence of the alternatively spliced HLA-G mRNA forms in human mononuclear cells from peripheral blood and umbilical cord blood. Hum Immunol. 43(3):237–241. 7558942 10.1016/0198-8859(95)00008-r

[CIT0073] Klein J. 1979 The major histocompatibility complex of the mouse. Science. 203(4380):516–521. 104386 10.1126/science.104386

[CIT0074] Klein J. 1986 Natural history of the major histocompatibility complex. 99th ed Wiley, New York, USA. ISBN 978-0471809531.

[CIT0075] Klein JAC, Schönbach 1993 Nomenclature for the major histocompatibility complexes of different species: a proposal In: Solheim BG, Ferrone S, Möller E, editors, The HLA system in clinical transplantation. Springer-Verlag, Berlin, Heidelberg, Germany, p. 16–37. ISBN 978-3-642-77508-6..

[CIT0076] Kovats S, Main E, Librach C, Stubblebine M, Fisher S, DeMars R. 1990 A class I antigen, HLA - G, expressed in human trophoblasts. Science. 248(4952):220–223. 2326636 10.1126/science.2326636

[CIT0077] Kutteh WH. 1996 Immunology of multiple endocrinopathies associated with premature ovarian failure. Endocrinologist. 6(6):462–466.

[CIT0078] Kutteh WH, Stanic AK, Schust DJ. 2019 Immunology and reproduction In: Strauss J, Barbier R, editors, Yen and Jaffe's Reproductive Endocrinology, 8^th^ ed. Physiology, pathophysiology, and clinical management. Elsevier, Philadelphia, PA, USA, p. 301–321. e3.ISBN 9780323479127.

[CIT0079] Larocca L, Ramhorst R, Roca V, Calafat M, Aisemberg J, Franchi A, Perez Leiros C. 2008 Neuroimmune-endocrine interactions during early pregnancy in an autoimmune context: focus on macrophage activation. Neuroimmunomodulation. 15(1):84–90. 18667804 10.1159/000135628

[CIT0080] Laškarin G, Kammerer U, Rukavina D, Thomson AW, Fernandez N, Blois SM. 2007 Antigen-presenting cells and materno-fetal tolerance: an emerging role for dendritic cells. Am J Reprod Immunol. 58(3):255–267. 17681042 10.1111/j.1600-0897.2007.00511.x

[CIT0081] Laškarin G, Redžović A, Vlastelić I, Haller H, Sršen-Medančić S, Solinas G, Rukavina D. 2011 Tumor-associated glycoprotein (TAG-72) is a natural ligand for the C-type lectin-like domain that induces anti-inflammatory orientation of early pregnancy decidual CD1a + dendritic cells. J Reprod Immunol. 88(1):12–23. 21172564 10.1016/j.jri.2010.10.001

[CIT0082] Lazarević M, Ejdus L, Hudina V, Miletić V. 1994 The influence of bovine seminal plasma fractions obtained by DEAE Sephacel chromatography on bovine lymphocyte blastogenesis. Acta Vet (Belgrade). 44(2-3):63–70.

[CIT0083] Lazarević M, Ejdus L, Rosić G. 1992 The influence of bovine seminal plasma, egg yolk extender and their mixture on bovine lymphocyte blastogenesis. Acta Vet (Belgrade). 42(4):227–236.

[CIT0084] Lazarević M, Jaćević V. 1998 Spermagglutinins and fertility of heifers and cows. Vet Glasnik. 52(7-8):345–356.

[CIT0085] Lazarević M, Kirovski D, Fratrić N, Milanović S, Jakovljević G, Milovanović A. 2002 The presence of naturally occurring antisperm antibodies in the sera of prepubertal calves. Acta Vet (Belgrade). 52(5-6):311–318.

[CIT0086] Lazarević M, Milanović S, Kirovski D, Magaš V. 2004a The local immune response in cows to sperm and semen extender antigens. Med Vet Vet Med Timisoara. 37:671–678.

[CIT0087] Lazarević M, Milanović S, Kirovski D, Milovanović A. 2003 Antisperm antibodies of the Ig A class in the cervical mucus and sera of artificially inseminated cows. Acta Vet (Belgrade). 53(5-6):311–319.

[CIT0088] Lazarević M, Miletić V. 1990 Bovine seminal plasma inhibition of complement. Period Biol. 92(Supp 3):84.

[CIT0089] Lazarević M, Milovanović A, Jovičin M, Šabanović M, Suljkanović A. 2006 Cutaneous basophil hypersensitivity reaction to phytohemagglutinin in heifers and cows with different reproductive results. Med Vet. Veterinary Medicine, Timisoara. 39:670–678.

[CIT0090] Lazarević M, Milovanović A, Milanović S, Kirovski D, Ilić V. 2004b Cutaneous basophil hypersensitivity reaction to phytohemagglutinin in repeat breeder cows. Acta Vet. (Belgrade). 54(5-6):337–346.

[CIT0091] Lazarević M, Skibinski G, Kelly RW, James K. 1995 Immunomodulatory effects of extracellular secretory vesicles isolated from bovine semen. Vet Immunol Immunopathol. 44(3-4):237–250. 7747404 10.1016/0165-2427(94)05320-r

[CIT0092] Lazarević M, Suljkanović A, Mickov A, Šabanović M, Paprikić N, Mlinar S. 2013 The influence of antisperm IgG and IgA antibodies from cows sera and cervical mucus on bull sperm motility. Acta Vet (Beogr)). 63(5-6):499–511.

[CIT0093] Lazarević M. 1996 Vesiculosomes – extracellular immunosuppressive particles in seminal plasma of bulls. Vet Glasnik. 50(9):663–670.

[CIT0094] Lazarević M, Miletić V. 1993 Bovine seminal plasma inhibition of immune complex precipitation. Acta Vet (Belgrade). 43(2-3):105–112.

[CIT0095] Loureiro B, Block J, Favoreto MG, Carambula C, Pennington KA, Ealy AD, Hansen PJ. 2011b Consequences of conceptus exposure to colony stimulating factor 2 on survival, elongation, IFN γ secretion and gene expression. Reproduction. 141(5):617–624. 21339286 10.1530/REP-10-0511

[CIT0096] Loureiro B, Oliveira LJ, Favoreto MG, Hansen PJ. 2011a Colony-stimulating factor 2 inhibits induction of apoptosis in the bovine preimplantation embryo. Am J Reprod Immunol. 65(6):578–588. 21223422 10.1111/j.1600-0897.2010.00953.x

[CIT0097] Lazary S. 1980 Equine leucocyte antigen (ELA) system Proc. 2^nd^ Symp. of Vet. Lab. Diagnosticians, Lucerne, Switzerland, p. 97–100.

[CIT0098] Lewis JE, Coulam CB, Breanndan Moore S. 1986 Immunological mechanisms in the maternal- fetal relationship. Mayo Clin Proc. 61(8):655–665. 3523061 10.1016/s0025-6196(12)62031-x

[CIT0099] Little CC. 1941 Chapter 24: The genetics of tumor transplantation In: Snell GD, editors. Biology of the laboratory mouse. Blakiston, Philadelphia; p. 279–309.

[CIT0100] Lugaro G, Manera E, Casellato MM, Bernasconi G, Sansò M, Fachini G. 1984 Bovine seminal plasma contains a low-molecular-weight factor that inhibits RNA synthesis . Arch Androl. 13(2-3):261–267. 6085717 10.3109/01485018408987525

[CIT0101] Lynch VJ, Nnamani MC, Kapusta A, Brayer K, Plaza SL, Mazur EC, Emera D, Sheikh SZ, Grützner F, Bauersachs S, et al. 2015 Ancient transposable elements transformed the uterine regulatory landscape and transcriptome during the evolution of mammalian pregnancy. Cell Rep. 10(4):551–561. 25640180 10.1016/j.celrep.2014.12.052 PMC4447085

[CIT0102] Maeda Y, Ohtsuka H, Tomioka M, Oikawa M. 2013 Effect of progesterone on Th1/Th2/Th17 and regulatory T cell-related genes in peripheral blood mononuclear cells during pregnancy in cows. Vet Res Commun. 37(1):43–49. 23203561 10.1007/s11259-012-9545-7

[CIT0103] Mahi-Brown CA, Yule TD, Tung KSK. 1987 Adoptive transfer of murine autoimmune orchitis to naive recipients with immune lymphocytes. Cell Immunol. 106(2):408–419. 2952288 10.1016/0008-8749(87)90183-3

[CIT0104] Mann T. 1974 Secretory function of the prostate, seminal vesicle and other male accessory organs of reproduction. J Reprod Fertil. 37(1):179–188. 4593605 10.1530/jrf.0.0370179

[CIT0105] Makrigiannakis A, Petsas G, Toth B, Relakis K, Jeschke U. 2011 Recent advances in understanding immunology of reproductive failure. J Reprod Immunol. 90(1):96–104. 21683452 10.1016/j.jri.2011.03.006

[CIT0106] Markert UR, Morales-Prieto DM, Fitzgerald JS. 2011 Understanding the link between the IL-6 cytokine family and pregnancy: implications for future therapeutics. Expert Rev Clin Immunol. 7(5):603–609. 21895473 10.1586/eci.11.60

[CIT0107] Marković F, Pavičić Ž, Samardžija M, Valpotić I, Gereš D, Dobranić T, Gračner D, Horvat – Marković R, Resanović R. 2008 Preliminary tests for the homeopathic preparation Traumeel^®^ on immune parameters and values of sperm quality in boars. Tierȕrztl Umsch. 63(6):312–321.

[CIT0108] Marković F, Pavičić Ž, Valpotić I, Mihaljević Ž, Grizelj J, Dobranić T, Horvat-Marković R, Ostović M, Đuričić D, Mršić G, et al. 2010 Parapoxvirus ovis preparation reduces the concentration of cortisol and enhances the bull sperm quality. Tierȕrztl Umsch. 65(6):224–228.

[CIT0109] Marshburn PB, Kutteh WH. 1994 The role of antisperm antibodies in infertility. Fertil Steril. 61(5):799–811. 8174713 10.1016/s0015-0282(16)56687-4

[CIT0110] Matoušek J. 1985 Biological and immunological roles of proteins in the sperm of domestic animals. Anim Reprod. Sci. 8(1-2):1–40.

[CIT0111] Matoušek J, Stanek R, Riha J. 1985 Some immunological phenomena in normal pregnant and embryo transferred cows. Acta Vet (Beograd). 35:17–26.

[CIT0112] Matoušek J, Stanek R, Hruba A. 1988 Immunosuppressive substances in bovine cervical mucus. Acta Vet (Beograd). 38:43–46.

[CIT0113] Medawar PB. 1946 Immunity to homologous grafted skin. II. The relationship between the antigens of blood and skin. Brit J Exp Path. 27(1):15–24. 20989196 PMC2065734

[CIT0114] Medawar PB. 1953 Some immunological and endocrinological problems raised by the evolution of viviparity in vertebrates. Symp Soc Exp Biol. 7:320–338.

[CIT0115] Medawar PB. 1961 Immunological tolerance. Science. 133(3449):303–306. 13768822 10.1126/science.133.3449.303

[CIT0116] Menge AC. 1967 Induced infertility in cattle by iso-immunization with semen and testis. J Reprod Fertil. 13(3):445–456. 6067509 10.1530/jrf.0.0130445

[CIT0117] Millot P. 2009 The major histocompatibility complex of sheep (OLA) and two minor loci. Anim Blood Grps Biochem Genet. 9(2):115–121. 10.1111/j.1365-2052.1978.tb01421.x 742735

[CIT0118] Milovanović A, Lazarević M, Milanović S, Kirovski D, Jovičin M. 2005 Open days period and antispermatozoal antibodies in artificially inseminated cows. Acta Vet (Beogr).). 55(5-6):449–460.

[CIT0119] Milovanović A, Lazarević M, Kirovski D, Jovičin M, Barna T. 2008 Antibody titer against spermatozoal antigens of bulls in blood sera of cows and heifers with different number of insemination. Arhiv Bet Med. 1(2):53–63.

[CIT0120] Ober C, Van Der Ven K. 1997 Immunogenetics of reproduction: an overview. Curr Top Microbiol Immunol. 222:1–23. 9257483 10.1007/978-3-642-60614-4_1

[CIT0121] Ober CL, Hauck WW, Kostyu DD, O’Brien E, Elias S, Simpson JL, Martin AO. 1985 Adverse effects of HLA-DR sharing on fertility: a cohort study in a human isolate. Fertil Steril. 44(2):227–232. 3860403

[CIT0122] Osborne L, Brar MA, Klein SL. 2019 The role of Th17 cells in the pathophysiology of pregnancy and perinatal mood and anxiety disorders. Brain Behavior Immunity. 76:7–16. 10.1016/j.bbi.2018.11.015 PMC6359933 30465878

[CIT0123] Payne R. 1957 Leukocyte agglutinins in human sera; correlation between blood transfusions and their development. AMA Arch Intern Med. 99(4):587–606. 13410165 10.1001/archinte.1957.00260040087010

[CIT0124] Peterson HB, Lamemel J, Stites PD, Brooks FG. 1980 Human seminal plasma inhibition of complement. J Lab Clin Med. 96:582–583. 6775032

[CIT0125] Pinget GV, Corpuz TM, Stolp J, Lousberg EL, Diener KR, Robertson SA, Sprent J, Webster KE. 2016 The majority of murine gammadelta T cellsat the maternal fetal interface in pregnancy produce IL-17. Immunol Cell Biol. 94(7):623–630. 27241697 10.1038/icb.2016.48

[CIT0126] Pongcharoen S, Somran J, Sritippayawan S, Niumsup P, Chanchan P, Butkhamchot P, Tatiwat P, Kunngurn S, Searle RF. 2007 Interleukin-17 expression in the human placenta. Placenta. 28(1):59–63. 16549200 10.1016/j.placenta.2006.01.016

[CIT0127] Potočnjak D, Pavičić Ž, Valpotić H, Popović M, Bedrica L, Vijtiuk N, Vlahović K, Mrljak V, Barić-Rafaj R. 2006 Reproductive performance of late pregnant gilts treated with Baypamun^©^ before farrowing. Acta Vet Brno. 75(3):373–377.

[CIT0128] Prakash C, Coutinho A, Möller G. 1976 Inhibition of in vitro immune responses by a fraction from seminal plasma. Scand J Immunol. 5(1-2):77–85. 10.1111/j.1365-3083.1976.tb02994.x 131370

[CIT0129] Price JR, Boettcher B. 1979 The present of complement in human cervical mucus and its possible relevance to infertility to women with complement- dependent sperm immobilizing antibodies. Fertil Steril. 32(1):61–66. 456632 10.1016/s0015-0282(16)44117-8

[CIT0130] Raghupathy R, Shaha C, Gupta SK. 1990 Autoimmunity to sperm antigens. Curr Opin Immunol. 2(5):757–760. 10.1016/0952-7915(90)90046-j 2701980

[CIT0131] Ratner LE, Hadley GA, Hanto DW, Mohanakumar T. 1991 Immunology of renal allograft rejection. Arch Pathol Lab Med. 115(3):283–287. 2001169

[CIT0132] Redžović A, Laškarin G, Dominović M, Haller H, Rukavina D. 2013 Mucins help to avoid alloreactivity at the maternal fetal interface. Clin Dev Immunol. 2013:542152. 23864879 10.1155/2013/542152 PMC3705806

[CIT0133] Robertson SA, Guerin LR, Moldenhauer LM, Hayball JD. 2009 Activating T regulatory cells for tolerance in early pregnancy – the contribution of seminal fluid. J Reprod Immunol. 83(1-2):109–116. 19875178 10.1016/j.jri.2009.08.003

[CIT0134] Rukavina D, Štrbo N, Sotošek V, Bedenicki I, Laškarin G. 1998 Recent advances in immunology of early pregnancy. Gynecol Perinatol. 7(2):77–83.

[CIT0135] Saito S, Nakashima A, Shima T, Ito M. 2010 Th1/Th2/Th17 and regulatory T-cell paradigm in pregnancy . Am J Reprod Immunol. 63(6):601–610. 20455873 10.1111/j.1600-0897.2010.00852.x

[CIT0136] Saito S, Nakashima A, Ito M, Shima T. 2011 Clinical implication of recent advances in our understanding of IL-17 and reproductive immunology. Expert Rev Clin Immunol. 7(5):649–657. 21895477 10.1586/eci.11.49

[CIT0137] Samardžija M, Marković F, Pavičić Ž, Gereš D, Valpotić I, Dobranić T, Horvat-Marković R, Gračner D, Lipar M, Radišić B. 2008 The evaluation of levamisole on immunostimulation and sperm quality in boars. Tierȕrztl Umsch. 63(9):480–495.

[CIT0138] Schifferly JA, Xao P, Peters DK. 1982 Complement mediated inhibition of immune complex precipitation, I Role of the classical and alternative pathway. Clin Exp Immunol. 47(3):555–562. 6979442 PMC1536444

[CIT0139] Saratsis PH, Alexopoulos C, Mavromatis J, Tsinas AC, Kyriakis SC. 1999 The use of an immunomodulator to enhance the reproductive performance of gilts transported for long distances from breeding to commercial units. Reprod Domest Anim. 34(2):67–70.

[CIT0140] Schmidt CM, Orr HT. 1993 Maternal/fetal interactions: the role of the MHC class I molecule HLA-G. Crit Rev Immunol. 13(3-4):207–224. 8110376

[CIT0141] Schuberth HJ, Taylor U, Zerbe H, Waberski D, Hunter R, Rath D. 2008 Immunological responses to semen in the female genital tract. Theriogenology. 70(8):1174–1181. 18757083 10.1016/j.theriogenology.2008.07.020

[CIT0142] Schumacher A. 2017 Human chorionic gonadotropin as a pivotal endocrine immune regulator initiating and preserving fetal tolerance. IJMS. 18(10):2166–2175. 10.3390/ijms18102166 PMC5666847 29039764

[CIT0143] Sharma S, Dhaliwal GS. 2010 Escherichia coli lipopolysaccharide-induced immunomodulation along with oxytocin administration after mating as a treatment protocol for persistent endometritis in mares. J. Equine Vet. Sci. 30(5):259–265.

[CIT0144] Skibinski G, Kelly RW, Harrison C, Mc Millan AL, James K. 1992a Relative immunosuppressive activity of human seminal prostaglandins. J Reprod Immunol. 22(2):185–195. 1501205 10.1016/0165-0378(92)90015-v

[CIT0145] Skibinski G, Kelly RW, Harkis D, James K. 1992b Immunosuppression by human seminal plasma-extracellular organelles (prostasomes) modulate activity of phagocytic cells. Am J Reprod Immunol. 28(2):97–103. 1337434 10.1111/j.1600-0897.1992.tb00767.x

[CIT0146] Snell GD. 1948 Methods for the study of histocompatibility genes. J Genet. 49(2):87–108. 18893744 10.1007/BF02986826

[CIT0147] Snell GD, Higgins GF. 1951 Alleles at the histocompatibility-2 locus in the mouse as determined by tumor transplantation. Genetics. 36(3):306–310. 14840651 10.1093/genetics/36.3.306 PMC1209522

[CIT0148] Stites PD, Erickson PR. 1975 Suppressive effect of seminal plasma on lymphocyte activation. Nature. 253(5494):727–729. 123041 10.1038/253727a0

[CIT0149] Taş M, Bacinoglu S, Cirit U, Ozdaş OB, Ak K. 2007 Relationship between bovine fertility and the number of spermatozoa penetrating the cervical mucus within straws. Anim Reprod Sci. 101(1-2):18–27. 16971070 10.1016/j.anireprosci.2006.08.020

[CIT0150] Telleria CM, Ou J, Sugino N, Ferguson S, Gibori G. 1998 The expression of interleukin-6 in the pregnant rat corpus luteum and its regulation by progesterone and glucocorticoid. Endocrinology. 139(8):3597–3605. 9681513 10.1210/endo.139.8.6132

[CIT0151] Toshitani K, Braud V, Browning MJ, Murray N, Mcmichael AJ, Bodmer WF. 1996 Expression of a single-chain HLA class I molecule in a human cell line: presentation of exogenous peptide and processed antigen to cytotoxic T lymphocytes. Proc Natl Acad Sci USA. 93(1):236–240. 8552612 10.1073/pnas.93.1.236 PMC40213

[CIT0152] Tissot RG, Cohen C. 1972 Histocompatibility in the rabbit: Idenification of the major locus. Tiss Antig. 2(4):267–279. 4586481

[CIT0153] Tripathi D, Sharma NC, Singh SK, Gupta LK. 1999 Identification of bovine sperm specific polypeptides reactive with antisperm antibodies. Indian J Exp Biol. 37(7):655–661. 10522153

[CIT0154] Trowsdale J, Hanson I, Mockridge I, Beck S, Townsend A, Kelly A. 1990 Sequences encoded in the class II region of the MHC related to the 'ABC' superfamily of transporters. Nature. 348(6303):741–743. 2259383 10.1038/348741a0

[CIT0155] Vaiman M, Chr R, La Fage P, Ametau J, Nizza P. 1970 Evidence for a histocompatibility system in swine (SLA). Transplantation. 10:155–164. 4915555 10.1097/00007890-197008000-00002

[CIT0156] Van Dam RH. 1981 Definition and biological significance of the major histocompatibility system (MHS) in man and animals. Vet Immunol Immunopathol. 2(6):517–539.

[CIT0157] Van Dam RH, D'amaro J, Van Kooten PJS, Van Der Donk JA, Goudswaard J. 1979 The histocompatibility complex GLA in the goat. Anim Blood Grps Biochem Genet. 10(2):121–124. 10.1111/j.1365-2052.1979.tb01015.x 292353

[CIT0158] Van Rood JJ, Van Leeuwen A, Eernisse JG. 1958 Leucocyte antibodies in sera from pregnant women. Nature. 181(4625):1735–1736. 13566127 10.1038/1811735a0

[CIT0159] Van Rood JJ, Claas FHJ. 1990 The influence of allogeneic cells on the human T and B cell repertoire. Science. 248(4961):1388–1393. 1972596 10.1126/science.1972596

[CIT0160] Veljković-Vujaklija D, Mulac-Jeričević B, Laškarin G, Dominović M, Tijanić T, Sršen Medančić S, Rukavina D. 2011 Immunoregulation by cytolytic pathways: mucins and progesteron at the maternal-fetal interface. Adv Neuroimmune Biol. 2(1,2):31–40.

[CIT0161] Veljković-Vujaklija D, Sučić S, Gulić T, Dominović M, Rukavina D. 2012 Cell death mechanisms at the maternal-fetal interface: insights into the role of granulysin. Clin Dev Immunol. 2012:180272article ID 180272, 8 pages. 21912564 10.1155/2012/180272 PMC3170798

[CIT0162] Verma OP, Kumar R, Kumar A, Chand S. 2012 Assisted reproductive techniques in farm animal - from artificial insemination to nanobiotechnology. Vet World. 5(5):301–310.

[CIT0163] Vickram AS, Samad HA, Latheef SK, Chakraborty S, Dhama K, Sridharan TB, Sundaram T, Gulothungan G. 2020 Human prostasomes an extracellular vesicle - Biomarkers for male infertility and prostrate cancer: The journey from identification to current knowledge. Int J Biol Macromol. 146:946–958. 31730983 10.1016/j.ijbiomac.2019.09.218

[CIT0164] Vikrant J, Ranjna C, Amrit B, Vinod G, Shabaz D. 2016 Status of antisperm antibodies in blood serum and cervical mucus of artificially inseminated crossbreed cows in relation to infertility. Anim Sci Rep. 10(1):34–40.

[CIT0165] Voisin GA, Delaunay A, Barber M. 1951 Sur des lesions testicularies provoquees ches le cobaye par iso-sensibilisation et auto-sensibilisation. Ann Immunol Inst Pasteur. 81:48–63. 14857453

[CIT0166] Vukotić M, Pavlović M. 1981 Selective adherence of FITC – conjugated seminal plasma to cell chromatin. Acta Vet (Beograd). 31:87–93.

[CIT0167] Vukotić M, Pavlović M. 1986 The possible immunological consequence of artificial insemination of cows. Period biol. 88:471–472.

[CIT0168] Wattegedera S, Rocchi M, Sales J, Howard CJ, Hope JC, Entrican G. 2008 Antigen-specific peripheral immune responses are unaltered during normal pregnancy in sheep. J Reprod Immunol. 77(2):171–178. 17826845 10.1016/j.jri.2007.07.003

[CIT0169] Witkin SS. 1988 Mechanisms of active suppression on the immune response to spermatozoa. Am J Reprod Immunol Micribiol. 17(2):61–64. 10.1111/j.1600-0897.1988.tb00204.x 2973252

[CIT0170] Wu HX, Jin LP, Xu B, Liang SS, Li DJ. 2014 Decidual stromal cells recruit Th17 cells into decidua to promote proliferation and invasion of human trophoblast cells by secreting IL-17. Cell Mol Immunol. 11(3):253–262. 24633013 10.1038/cmi.2013.67 PMC4085486

[CIT0171] Yang Y, Chu W, Geraghty DE, Hunt JS. 1996 Expression of HLA-G in human mononuclear phagocytes and selective induction by IFN-gamma. J Immunol. 156(11):4224–4231. 8666791

[CIT0172] Yelavarth K, Fishback LLI, Hunt JA. 1991 Analysis of HLA-G mRNA in human placental and extraplacental membrane cells by in situ hybridization. J. Immunol. 146(8):2847–2854. 2016528

[CIT0173] Yitbarek MB, Regasa F. 2014 Reproductive immunization of domestic and wild animals: review. Int. J. Sci. Technol. Res. 3(4):399–412.

[CIT0174] Young SL. 2016 Reproductive immunology: checkered past and bright future. Fertil. Steril. 106(3):497–498. 27479270 10.1016/j.fertnstert.2016.07.1090 PMC5010996

[CIT0175] Yuhki N, O'Brien SJ. 1988 Molecular characterization and genetic mapping of class I and class II MHC genes of the domestic cat. Immunogenetics. 27(6):414–425. 2897330 10.1007/BF00364427

[CIT0176] Zinkernagel RM, Doherty PC. 1974a Restriction of in vitro T cell-mediated cytotoxicity in lymphocytic choriomeningitis within a syngeneic or semiallogeneic system. Nature. 248(5450):701–702. 4133807 10.1038/248701a0

[CIT0177] Zinkernagel RM, Doherty PC. 1974b Immunological surveillance against altered self components by sensitised T lymphocytes in lymphocytic choriomeningitis. Nature. 251(5475):547–548. 4547543 10.1038/251547a0

