# Correction notice

**DOI:** 10.1080/01652176.2021.1884347

**Published:** 2021-04-13

**Authors:** M. Samardžija, M. Lojkić, N. Maćešić, H. Valpotić, I. Butković, J. Šavorić, I.Žura Žaja, D. Leiner, D. Đuričić, F. Marković, P. Kočila, Ž. Vidas, M. Gerenčer, A. Kaštelan, A. Milovanović, M. Lazarević, D. Rukavina, I. Valpotić

**Affiliations:** a Clinic for Obstetrics and Reproduction of Animals, Veterinary Faculty University of Zagreb, Zagreb, Croatia; b Department for Animal Nutrition and Dietetics, Veterinary Faculty University of Zagreb, Zagreb, Croatia; c Department for Physiology and Radiobiology, Veterinary Faculty University of Zagreb, Zagreb, Croatia; d Department of Anatomy, Histology and Embriology, Veterinary Faculty University of Zagreb, Zagreb, Croatia; e Veterinary Practice, Đurđevac, Croatia; f Belupo d.d. Danica, Koprivnica, Croatia; g Animal Feed Factory, Cakovec, Croatia; h Faculty of Medicine, Department of Urology, Josip Juraj Strossmayer University of Osijek, Osijek, Croatia; i Croatian Academy of Sciences and Arts, Croatian Academy of Sciences and Arts, Zagreb, Croatia; j Department of Reproduction, Veterinary Scientific Institute, Novi Sad, Serbia; k Department for Physiology and Biochemistry, Faculty of Veterinary Medicine, University of Belgrade, Belgrade, Serbia; l Department of Cellular Immunology, Baxter Hyland Immuno, Vienna, Austria; m Department of Biology, Veterinary Faculty University of Zagreb, Zagreb, Croatia

**Article title:** Reproductive immunology in viviparous mammals: evolutionary paradox of interactions among immune mechanisms and autologous or allogeneic gametes and semiallogeneic fetuses

**Authors:** Samardžija M, M. Lojkić, N. Maćešić, H. Valpotić, I. Butković, J. Šavorić, I. Žura Žaja, D. Leiner, D. Đuričić, F. Marković, P. Kočila, Ž. Vidas, M. Gerenčer, A. Kaštelan, A. Milovanović, M. Lazarević, D. Rukavina, I. Valpotić

**Journal:** Veterinary Quarterly

**DOI:** 10.1080/01652176.2020.1852336

In the original manuscript the affiliations for the authors M. Gerenčer, A. Kaštelan, A. Milovanović, M. Lazarević, D. Rukavina, I. Valpotić are set incorrectly. This has now been corrected as shown below.

M. Samardžija^a^, M. Lojkić^a^, N. Maćešić^a^, H. Valpotić^b^, I. Butković^a^, J. Šavorić^a^, I. Žura Žaja^c^, D. Leiner^d^, D. Đuričić^e^, F. Marković^f^, P. Kočila^g^, Ž. Vidas^h^, M. Gerenčer^l^, A. Kaštelan^i^, A. Milovanović^j^, M. Lazarević^k^, D. Rukavina^i^ and I. Valpotić^m^

^a^Clinic for Obstetrics and Reproduction of Animals, Veterinary Faculty University of Zagreb, Zagreb, Croatia; ^b^Department for Animal Nutrition and Dietetics, Veterinary Faculty University of Zagreb, Zagreb, Croatia; ^c^Department for Physiology and Radiobiology, Veterinary Faculty University of Zagreb, Zagreb, Croatia; ^d^Department of Anatomy, Histology and Embriology, Veterinary Faculty University of Zagreb, Zagreb, Croatia; ^e^Veterinary Practice, Đurđevac, Croatia; ^f^Belupo d.d. Danica, Koprivnica, Croatia; ^g^Animal Feed Factory, Cakovec, Croatia; ^h^Faculty of Medicine, Department of Urology, Josip Juraj Strossmayer University of Osijek, Osijek, Croatia; ^i^Croatian Academy of Sciences and Arts, Croatian Academy of Sciences and Arts, Zagreb, Croatia; ^j^Department of Reproduction, Veterinary Scientific Institute, Novi Sad, Serbia; ^k^Department for Physiology and Biochemistry, Faculty of Veterinary Medicine, University of Belgrade, Belgrade, Serbia; ^l^Department of Cellular Immunology, Baxter Hyland Immuno, Vienna, Austria; ^m^Department of Biology, Veterinary Faculty University of Zagreb, Zagreb, Croatia

